# Identification of Sitogluside as a Potential Skin-Pigmentation-Reducing Agent through Network Pharmacology

**DOI:** 10.1155/2021/4883398

**Published:** 2021-09-23

**Authors:** Haoran Guo, Hongliang Zeng, Chuhan Fu, Jinhua Huang, Jianyun Lu, Yibo Hu, Ying Zhou, Liping Luo, Yushan Zhang, Lan Zhang, Jing Chen, Qinghai Zeng

**Affiliations:** ^1^Department of Dermatology, The Third Xiangya Hospital, Central South University, Changsha 410013, China; ^2^Institute of Chinese Materia Medica, Hunan Academy of Chinese Medicine, Changsha 410013, China

## Abstract

Many traditional Chinese medicines (TCMs) with skin-whitening properties have been recorded in the *Ben-Cao-Gang-Mu* and in folk prescriptions, and some literature confirms that their extracts do have the potential to inhibit pigmentation. However, no systematic studies have identified the specific regulatory mechanisms of the potential active ingredients. The aim of this study was to screen the ingredients in TCMs that inhibit skin pigmentation through a network pharmacology system and to explore underlying mechanisms. We identified 148 potential active ingredients from 14 TCMs, and based on the average “degree” of the topological parameters, the top five TCMs (*Fructus Ligustri Lucidi*, *Hedysarum multijugum Maxim.*, *Ampelopsis japonica*, *Pseudobulbus Cremastrae Seu Pleiones*, and *Paeoniae Radix Alba*) that were most likely to cause skin-whitening through anti-inflammatory processes were selected. Sitogluside, the most common ingredient in the top five TCMs, inhibits melanogenesis in human melanoma cells (MNT1) and murine melanoma cells (B16F0) and decreases skin pigmentation in zebrafish. Furthermore, mechanistic research revealed that sitogluside is capable of downregulating tyrosinase (TYR) expression by inhibiting the ERK and p38 pathways and inhibiting TYR activity. These results demonstrate that network pharmacology is an effective tool for the discovery of natural compounds with skin-whitening properties and determination of their possible mechanisms. Sitogluside is a novel skin-whitening active ingredient with dual regulatory effects that inhibit TYR expression and activity.

## 1. Introduction

Melanin not only determines the color of human skin, eyes, and hair but also plays a key role in camouflage, mimicry, social communication, and protection from ultraviolet rays. However, when melanin is overproduced and abnormally distributed in the epidermis, it causes a variety of conditions, such as freckles and melasma, which can negatively affect one's perceived beauty and social confidence [1]. Melanin is the final product of L-tyrosine following multiple steps of enzymatic reactions and is synthesized in melanocyte melanosomes [2]. In the skin, melanocytes are located in the basal layer of the epidermis and can produce and transfer mature melanosomes to keratinocytes, thus causing skin pigmentation [3]. TYR is the main rate-limiting enzyme in melanin production [4], and microphthalmia-associated transcription factor (MITF) is the main transcription factor that regulates the transcriptional expression of TYR [5].

Recently, studies have found that inflammatory responses play important roles in the regulation of melanogenesis [6]. For example, IL-18, produced by Langerhans cells, dendritic cells, and keratinocytes in the epidermis, can upregulate the expressions of TRP-1 (tyrosinase-related protein 1) and TRP-2 (tyrosinase-related protein 2) by activating the p38/MAPK (mitogen-activated protein kinase) and PKA (protein kinase A) pathways, thus promoting melanogenesis [7, 8]. TNF-*α* downregulates B16 melanin production mainly by activating NF-*κ*B [9]. PGe2 (prostaglandin e2), which is secreted by fibroblasts and keratinocytes, has been shown to stimulate the differentiation of dendritic cells and initiate TYR activity in melanocytes through the cAMP (cyclic adenosine monophosphate) and Plc (phospholipase c) signaling pathways [10]. PIH (postinflammatory hyperpigmentation) is a type of reactive skin melanization [11]. Various skin diseases, trauma, or cosmetic surgery often lead to pigmentation of the affected area, especially in people of color [12]. Patients with PIH usually bear immense psychological stress, which in severe cases, negatively affects their quality of life. Therefore, certain medicines that regulate inflammation are used to treat PIH and other diseases that cause hyperpigmentation. For example, resveratrol, which reduced the inflammatory damage in HaCaT cells [13], was found to inhibit melanin synthesis through extracellular signal-regulating of kinase 1/2 and signaling of phosphoinositide 3-kinase/Akt [14].

Whitening agents, such as hydroquinone (HQ) and vitamin C, are traditionally used to treat pigmentation [15]. HQ is a hydroxyphenol with a powerful inhibitory effect on melanogenesis. However, due to various side effects such as irritant or allergic contact dermatitis, nail discoloration, and impaired wound healing, the use of HQ is restricted [16, 17]. Vitamin C inhibits TYR by interacting with copper ions at TYR active sites, thereby decreasing melanogenesis. However, vitamin C is unstable in finished products. In addition, if the concentration of vitamin C is higher than 20%, it can cause skin irritation [18]. Therefore, natural ingredients from a wide range of sources that are safe, effective, highly stable, and easy to store are in high demand.

Recently, more TCM ingredients have been found to be effective in inhibiting skin pigmentation, such as apigenin and paeoniflorin [19–21]. The TCMs with whitening activity described in the *Ben-Cao-Gang-Mu* are widely used as secret recipes in China, and nowadays, they are confirmed to have the effect of inhibiting pigmentation. For example, the extract of *Hedysarum multijugum Maxim* (the rhizome of *Astragalus membranaceus (Fisch.) Bunge*) causes skin whitening by inhibiting the ERK pathway [22]. The dry extract of *Typhonii Rhizoma* (the rhizome of *Sauromatum giganteum (Engl.) Cusimano* and *Hett.*) can effectively inhibit the tyrosinase activity *in vitro* [23]. And evidence-based medicine has also confirmed that the extract of *Sapindi Mukorossi Semen* (the fruit of *Sapindus mukorossi Gaertn*.), which inhibits tyrosinase activity, has the potential to be used as a drug and cosmetic additive [24]. Some traditional folk prescriptions, although not compiled into a compendium, are also widely used. *San-Bai-Tang* (SBT) is a typical example. Ye et al. demonstrated that SBT could decrease melanin production by inhibiting the p38/MAPK signaling pathway and TYR activity [25]. Other TCMs described in some prescriptions have also been shown to have skin-whitening effects. *Coicis Semen* (the seed of *Coix aquatica Roxb.*) extract inhibits melanin production by downregulating MITF, TYR, TRP-1, and TRP-2 [26]. Moreover, a cell assay of TYR showed that *Ampelopsis japonica* (the rhizome of *Ampelopsis japonica (Thunb.) Makino*) inhibited the activity of mushroom TYR by more than 50% [27]. The whitening TCMs recorded in these ancient prescriptions have been proved to be effective by modern experimental techniques, but most of them are verified in the form of extracts, and the active ingredients that work are not clear. This aroused our interest in the active ingredients of TCMs with whitening effects recorded in ancient prescriptions.

Besides, TCMs or prescriptions have complex ingredients, diverse modes of action, and unknown active ingredients. In addition, the inconsistency of factors such as the quality of raw materials, processing methods, preparation techniques, compatibility of ingredients used in the composition, and dosage forms has a greater impact on the efficacy of TCMs [19, 28]. Therefore, the usage of certain TCMs with skin-whitening effects is restricted. It is thus urgent to identify active ingredients with potential whitening effects on the skin, through systematic screening to ensure the safety, efficacy, and stability of these ingredients.

In recent years, the application of system biology in the field of TCM has made great progress. The network pharmacology of TCM is a new cross-discipline that combines the pharmacology of TCMs with network science, system biology, computational science, and bioinformatics [29]. It fully understands the complexity between drugs, diseases, and biological systems from the perspective of the “complex-protein/gene-disease” network [30]. Network pharmacology is an effective and systematic tool to study the pharmacological action, mechanism, safety, and other aspects of herbal medicine, especially TCMs, and provides valuable insights for current drug discovery [31, 32].

## 2. Materials and Methods

### 2.1. Network Pharmacological Process

#### 2.1.1. Database Building for Chemical Ingredients

**Figure 1:**
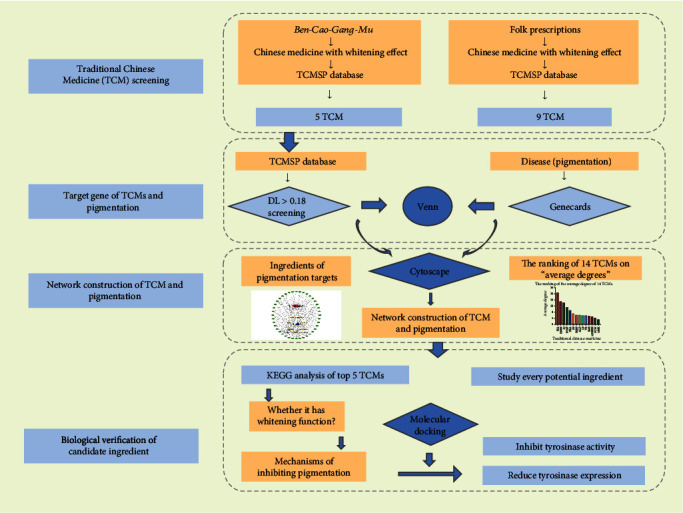
Flow chart of this study.

A flow chart of the studied experimental program is depicted in Figure 1. The TCMs with antipigmentation effects, described in the *Ben-Cao-Gang-Mu* and in folk prescriptions, were used for network pharmacological analysis. *Typhonii Rhizoma*, *Ricini Semen*, *Hedysarum multijugum Maxim.*, *Pseudobulbus Cremastrae Seu Pleiones*, and *Sapindi Mukorossi Semen*, described in the *Ben-Cao-Gang-Mu*, have whitening and yellow lowering properties and can remove facial spots and moles. *Poria cocos (Schw.) Wolf* (the sclerotium of *Smilax china L.*), *Paeoniae Radix Alba*, and *Atractylodes macrocephala Koidz* (the rhizome of *Atractylodes macrocephala Koidz*.) which are constituents of the SBT are known to improve skin texture and reduce chloasma and pigmentation. Other traditionally recorded herbs, such as *Bletilla striata (Thunb. Murray) Rchb.F.* (the rhizome of *Bletilla striata (Thunb.) Rchb.F.*), *Ampelopsis japonica*, *A. dahurica (Fisch.) Benth. Et Hook* (the rhizome of *Angelica dahurica (Hoffm.) Benth. and Hook.f. ex Franch. and Sav.*), *Atractylodes lancea (Thunb.) Dc.* (the rhizome of *Atractylodes lancea (Thunb.) DC.*), *Fructus Ligustri Lucidi*, and *Coicis Semen*, were also included in this study. Because of the large number of TCMs included in this study, multiple Chinese medicine database screenings will cause data processing confusion. We only use the Traditional Chinese Medicine Systems Pharmacology (TCMSP) database, a more commonly used database, for follow-up screening. The chemical ingredients of these TCMs were searched in TCMSP (updated on April 24, 2020) [33]. Drug-likeness (DL) and oral bioavailability (OB) are commonly used screening parameters. Topical or systemic medications are common treatments for skin pigmentation diseases; therefore, we only used DL as the screening criterion. Excellent complexes with DL > 0.18 were selected to increase the hit rate of drug candidates.

#### 2.1.2. Intersection between Target Genes of TCMs and Pigmentation-Associated Genes

The gene set associated with pigmentation was selected based on the GeneCards database (updated on April 24, 2020), from which targets with a gene-disease score > 1 were selected. Eventually, 6180 pigmentation-related targets were identified. The intersection between target genes of TCMs and pigmentation-associated genes was illustrated using a Venn diagram [34].

#### 2.1.3. Construction of an Active Compound-Target-Pathway Network

Active ingredients and regulated target genes were screened according to the results of the Venn diagram. The network was constructed using Cytoscape software [35] and was used to indicate the relationship between the active compounds of TCMs and the target genes of pigmentation. Each target gene regulated by an active ingredient in a TCM was considered one “degree.” The average “degree” topological parameters of each TCM were used to evaluate the importance of the selected TCMs. The compounds were ranked, and the top five TCMs were selected for key analyses. Furthermore, detailed information on the potential active ingredients reported to regulate pigmentation was analyzed.

#### 2.1.4. Kyoto Encyclopedia of Genes and Genome (KEGG) Enrichment Analysis of Target Genes Regulated by Potentially Effective TCMs

Target genes regulated by the top five TCMs were selected to determine KEGG target pathways and explore the possible mechanisms of potential effective ingredients in these TCMs against pigmentation. DAVID bioinformatics resources 6.8 was used to analyze the enrichment of KEGG target pathways. The gene symbols of the potential targets of the effective active ingredients were uploaded. The obtained gene identifiers were then downloaded and entered KOBAS3. The gene which adjusted *p* values less than 0.05 was selected.

#### 2.1.5. Molecular Docking Simulation

The 2D structures of sitogluside, arbutin, nicotinamide, HQ, kojic acid, and ascorbic acid were obtained from the PubChem database. The mechanical structures of the small molecules were optimized using ChemBio3D Ultra 14.0. The 3D crystal structure of mushroom TYR (PDB ID: 2Y9X, docking coordinates: *X* = −8.87, *Y* = −30.74, and *Z* = −41.52) was obtained from the Protein Data Bank (PDB) database [36]. The 3D crystal structures of six membrane receptors (opioid u receptor, PDB ID: 4DKI; melanocortin 1 receptor, PDB ID: 2L1J; endothelin B receptor, PDB ID: 5GLI; adrenergic *β* receptor, PDB ID: 4GBR; prostaglandin receptor, PDB ID: 6D26; stem cell factor receptor, PDB ID: 2EC8) were obtained from the PDB database. The protein structures were prepared using AutoDock Tools by removing water molecules, adding hydrogens, and creating zero-order bonds to metals and disulfide bonds. Lastly, molecular docking simulations were performed using AutoDock Vina 1.1.2 [37], and the results were analyzed using PyMoL 1.7.2.1.

### 2.2. Experimental Validation

#### 2.2.1. Chemicals and Antibodies

Sitogluside (purity ≥ 98%) was purchased from ChemFaces (#CFN98, 713, ChemFaces). Dimethylsulfoxide (DMSO) was purchased from Sigma-Aldrich. Neutral paraformaldehyde (4%) was purchased from Biosharp (Hefei, China). Kojic acid, the Fontana-Masson Stain Kit, sodium deoxycholate, and L-DOPA were purchased from Solarbio (Beijing, China). Dulbecco's modified Eagle's medium (DMEM) was purchased from Gibco (#C11995500BT, Gibco). Fetal bovine serum (FBS) and Cell Counting Kit-8 (CCK8) were purchased from BI (Kibbutz Beit-Haemek, Israel). PMA (12-O-tetradecanoyl phorbol-13-acetate, the ERK/MAPK activator) was purchased from APExBIO (#N2060, APExBIO). Anisomycin (the p38/MAPK activator) was purchased from MedChemExpress (HY-18982, MedChemExpress). Primary antibodies against ras-related protein Rab-27A (RAB27A) (#69295, CST), extracellular signal-regulated kinase (ERK) (#4695, CST), p-ERK (#4370, CST), c-Jun N-terminal kinase JNK (#9252, CST), p-JNK (#4668, CST), p38 (#8690, CST), p-p38 (#4511, CST), cAMP response element-binding protein CREB (#9107, CST), and p-CREB (#9198, CST) were purchased from Cell Signaling Technology. Primary antibodies against TRP1 (ab235447, Abcam), TRP2 (NBP1-56058, Novusbio), MITF (STJ94134, St. John's Laboratory), TYR (BS1484, Bioworld), and GAPDH (#AP0066, Bioworld) were purchased from Abcam, Novusbio, St. John's Laboratory, and Bioworld, respectively.

#### 2.2.2. Cell Culture and Treatment

MNT1 and B16F0, which are rich in melanosomes, are widely used to study melanogenesis [38]. Consequently, these two cell lines were chosen for subsequent experiments. MNT1 and B16F0 cells were cultured in 20% or 10% fetal bovine serum (Biological Industries, Israel) and 1% penicillin-streptomycin (Gibco) in DMEM. All cells were cultured in a wet incubator at 37°C and 5% CO_2_. Sitogluside was dissolved in DMSO, and the final concentration of DMSO was <0.1%. PMA and anisomycin were separately dissolved in DMSO, and the final concentration of DMSO was <0.1%.

#### 2.2.3. Zebrafish Culturing and Treatment

Zebrafish embryos and culture medium were purchased from EzeRinka Biotech (Nanjing, China). The experimental protocol was approved by the Ethics Committee of Third Xiangya Hospital of Central South University (No. 2021-S137). On the first day after hatching, the zebrafish were reared in a 12-well plate (10 per well), protected from light, and cultured at 37°C. The zebrafish were treated with different concentrations of sitogluside, and photographs were taken using an inverted microscope at 24 h intervals to record the changes in melanin in the tails of the zebrafish. After the pictures were taken, sitogluside solutions of different concentrations were readded and recorded continuously for one week.

#### 2.2.4. Cell Viability

Cell viability was assessed using a CCK8. MNT1 and B16F0 cells were plated in 96-well plates at a density of 2000 cells/well or 500 cells/well. The cells were treated with different concentrations of sitogluside (25, 50, 100, 200, 400, 600, and 800 *μ*M). After 24 h or 48 h, 10 *μ*L of CCK8 reagent was added to each well, and the cells were incubated at 37°C for 1 h. When the color of the medium turned orange, the culture was terminated, and a microplate reader (PerkinElmer EnVision xcite, UK) was used to measure the absorbance at 490 nm.

#### 2.2.5. Fontana-Masson Melanin Staining

Cells were cultured in a 6-well plate and treated with different concentrations of sitogluside for 2 days, and kojic acid as a positive control was added at 200 *μ*M. The cells were then placed in 4% paraformaldehyde for 15 min. After rinsing with water, the cells were incubated with Fontana ammonia-silver solution for 24 h in a dark chamber, rinsed with water, and then placed in hyposulphite for an additional 5 min. An inverted microscope was used to observe the melanin.

#### 2.2.6. Function Recovery Experiment

PMA, as a recognized activator of ERK/MAPK [39, 40], was tested for cytotoxicity through CCK8 experiment at different concentrations (6.25, 12.5, 25, 50, 100, 200, 400, and 800 ng/mL) for 24 hours. And after MNT1 and B16F0 cells were treated with different concentrations (25, 50, and 100 ng/mL) of PMA for 2 hours, the phosphorylation level of ERK was detected by western blotting experiment. At the selected concentration, the cells were treated with for 0, 1, 2, 4, 6, and 8 hours to detect the effective time of activation. After the cells were treated with sitogluside (200 *μ*M) and PMA (25 ng/mL) for 48 hours (retreated every 8 hours), the protein levels of TYR and p-ERK were detected. *Fontana-Masson melanin staining* detected the melanin content. Anisomycin [41] is the activator of p38/MAPK, the exploration concentration of which is 4, 8, 16, 32, 64, 128, 256, and 512 nM, and the other experimental methods are the same as PMA.

#### 2.2.7. TYR Activity Measurements

Cells were treated with different concentrations of sitogluside for 2 days. After digestion and centrifugation, the cells were counted and resuspended twice in phosphate-buffered saline (PBS). Next, 500 *μ*L of 0.5% sodium deoxycholate solution was added to each sample. The samples were incubated at 4°C for 10 min and then at 37°C for 15 min. 1 mL of 1% L-DOPA solution was added, following which 200 *μ*L was immediately pipetted into a 96-well plate. Subsequently, a multimode plate reader was used to measure the absorbance values (A0) at 475 nm, and the measurement was repeated after 30 min (A30). TYR activity was calculated as (A30-A0)/cell number.

#### 2.2.8. TYR Activity Probe

A near-infrared fluorescence probe for TYR activity based on resorufin was used to visualize activity in living cells, as described previously [42]. Briefly, MNT1 and B16F0 cells were cultured in a 6-well plate and treated with different concentrations of sitogluside for 2 days. A 10 *μ*M fluorescence probe was added, and after 4 h, the cells were replaced with PBS. After the red light was excited, a fluorescent inverted microscope was immediately used to take pictures.

#### 2.2.9. Protein Extraction and Western Blotting

After the cells were treated with different concentrations of sitogluside for 2 days, the total cellular protein was extracted using the RIPA Lysis Buffer (Thermo Fisher) and the Protease Inhibitor and Phosphatase Inhibitor cocktails (Roche). Protein concentration was determined using a BCA protein assay kit (KeyGEN Biotec). After blocking with 1% BSA, the primary antibodies against TYR, MITF, TRP1, TRP2, RAB27A, CREB, p-CREB, ERK, p-ERK, JUK, p-JUK, p38, and p-p38 were incubated with the membranes overnight at 4°C at 1 : 1000 and GAPDH at 1 : 3000. The membranes were washed with TBS-T and incubated with goat anti-rabbit secondary antibody at 1 : 3000 for 1 h. The binding antibody was detected by electrochemiluminescence (ECL).

#### 2.2.10. Statistical Analysis

SPSS22.0 software was used for statistical analysis, and Student's *t*-test or one-way analysis of variance (ANOVA) was used for multiple group comparisons. The Mann-Whitney *U* test was used for nonparametric data. *p* < 0.05 in all cases was considered statistically significant. ^∗∗∗^*p* < 0.001, ^∗∗^*p* < 0.01, and ^∗^*p* < 0.05.

## 3. Results

### 3.1. Composite Ingredients of TCMs

Fourteen TCMs, recorded in the *Ben-Cao-Gang-Mu* and in folk prescriptions, with antipigmentation effects were studied. A total of 372 Chinese medicine ingredients from the 14 TCMs were retrieved from the TCMSP database (supplementary Table S1).

### 3.2. Target Genes Regulated by TCMs and Related Pigmentation Genes

According to the target prediction system in the TCMSP database, a total of 834 target genes from the 14 TCMs were obtained. A total of 9431 genes related to pigmentation were retrieved from the GeneCards database, out of which, 6180 were selected and had a relevance score > 1.

### 3.3. Ranking of 14 TCMs and the Disease-Compound-Target Network

**Figure 2:**
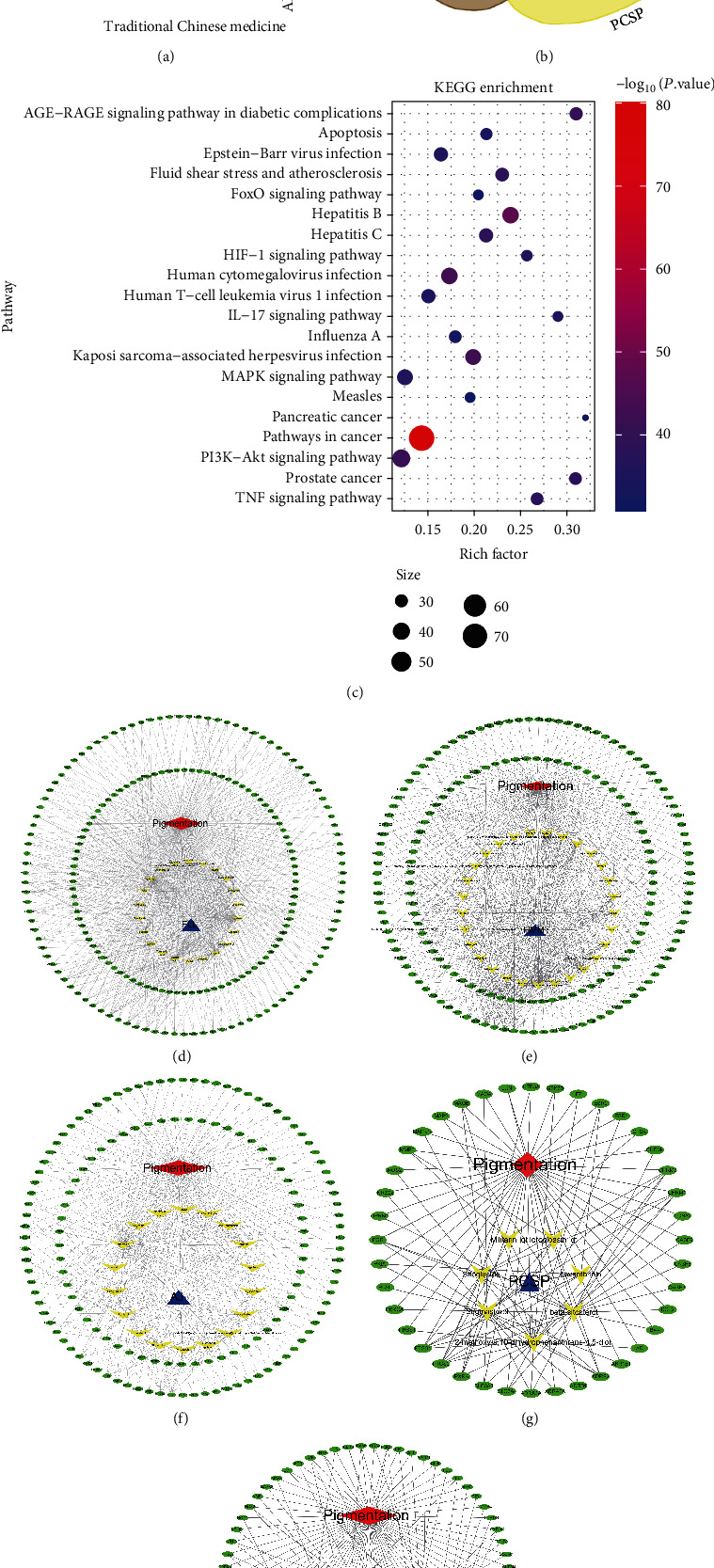
The detailed analysis of key TCMs. (a) The ranking of 14 TCMs, according to the “average degree” of each TCM. (b) The Venn diagram on the potential active ingredients of FLL, HMM., AJ, PCSP, and PRA. (c) The KEGG target pathways of the top 20 enriched signaling pathways in the top 5 TCMs. (d–f) The disease-compound-target network between FLL, HMM, AJ, PCSP, PRA, and pigmentation, respectively. The network is composed of nodes and lines. Red node indicates diseases. Blue node indicates herbs. Yellow nodes indicate potential Chinese medicine activity components, and green nodes indicate related target genes. Lines indicate their interactions. ADBEH: *A. dahurica (Fisch.) Benth. Et Hook*; AJ: *Ampelopsis japonica*; ALD: *Atractylodes lancea (Thunb.)Dc.*; AMK: *Atractylodes macrocephala Koidz.*; BSRF: *Bletilla striata (Thunb.Ex A.Murray) Rchb.F.*; CS: *Coicis Semen*; FLL: *Fructus Ligustri Lucidi*; HMM: *Hedysarum multijugum Maxim.*; KEGG: Kyoto Encyclopedia of Genes and Genome; PCSP: *Pseudobulbus Cremastrae Seu Pleiones*; PCW: *Poria Cocos (Schw.) Wolf.*; PRA: *Paeoniae Radix Alba*; RS: *Ricini Semen*; TR: *Typhonii Rhizoma*; SMS: *Sapindi Mukorossi Semen*; TCM: traditional Chinese medicine.

Using Cytoscape software, we took the intersection of the regulatory genes of each TCM and pigmentation-related genes and obtained the potential active ingredients and common target genes. Next, the disease-compound-target network between TCM and pigmentation was constructed. Based on this, we obtained the average “degree” of each active ingredient and ranked the TCMs according to their scores. According to the rank scores, the top 5 herbs were *Fructus Ligustri Lucidi*, *Hedysarum multijugum Maxim.*, *Ampelopsis japonica*, *Pseudobulbus Cremastrae Seu Pleiones*, and *Paeoniae Radix Alba* (Figure 2(a)). Through KEGG analysis of 220 target genes of the top 5 TCMs, 19.3% of genes were rich in the PI3K-Akt signaling pathway, 13.6% of genes were related to the TNF signaling pathway, and about 12.3% of genes were related to IL-17. In addition, 37 genes were involved in the MAPK pathway and 28 genes were involved in the HIF-1 pathway. These pathways are closely related to the regulation of melanin metabolism and skin photoaging. Among the top 20 enriched signaling pathways, inflammation-related pathways accounted for one-quarter of these (Figure 2(c)). Figures 2(d)–2(h) depict the disease-compound-target network between *Fructus Ligustri Lucidi*, *Hedysarum multijugum Maxim.*, *Ampelopsis japonica*, *Pseudobulbus Cremastrae Seu Pleiones*, *Paeoniae Radix Alba*, and pigmentation, respectively. According to the Venn diagram of the ingredients in the top five TCMs, we found that four TCMs contained sitogluside and beta-sitosterol (Figure 2(b), supplementary Table S2).

### 3.4. Potential Active Ingredients in Inhibiting Pigmentation

According to the disease-compound-target network, 148 ingredients related to pigmentation were obtained from 14 TCMs. Through a literature search, 25 of the 148 ingredients were reported to inhibit melanogenesis. Four active ingredients were reported as plant extracts that inhibit melanogenesis. Fourteen active ingredients were studied, and their derivatives were reported to regulate pigmentation. Interestingly, four ingredients were shown to promote melanogenesis, while the mechanism of six ingredients in regulating melanogenesis is still unclear. The remaining 95 potential Chinese medicinal ingredients have not yet been explored for the regulation of pigmentation. Detailed information on these ingredients is provided in supplementary Table S3.

### 3.5. In Vitro and In Vivo Effects of Sitogluside on Melanogenesis

**Figure 3:**
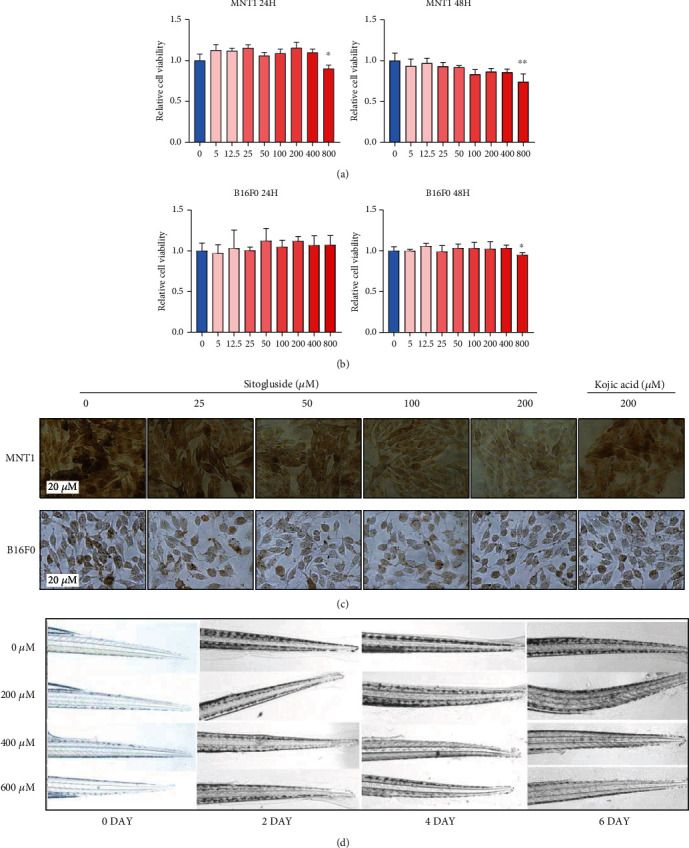
Sitogluside inhibits melanogenesis *in vitro* and *in vivo*. (a, b) CCK8 assay detects the cell viability of MNT1 and B16F0 cells treated with different concentrations of sitogluside. (c) Fontana-Masson melanin staining detects the melanin content. (d) Sitogluside can effectively inhibit the pigmentation of zebrafish.

After the above screening process, we found that four TCMs in the top TCMs contained sitogluside and *β*-sitosterol. *β*-Sitosterol has been reported to inhibit alpha-melanocyte-stimulating hormone (*α*-MSH), stimulating melanogenesis via the p38 signaling pathway in B16F10 melanoma cells [43]. However, the antipigmentation effects of sitogluside and its specific mechanism have not been reported. Therefore, we chose sitogluside as a drug candidate for these studies. The CCK8 assay was performed to explore the potential toxic concentrations of sitogluside in MNT1 and B16F0 cells. The results showed that sitogluside had no obvious effect on the proliferation of the cells (Figures 3(a) and 3(b)) at concentrations below 400 *μ*M. Melanin staining conducted using 25, 50, 100, and 200 *μ*M sitogluside showed that sitogluside can reduce melanin content in MNT1 and B16F0 cells in a dose-dependent manner (Figure 3(c)). Furthermore, sitogluside treatment significantly inhibited skin pigmentation in zebrafish in a time- and dose-dependent manner (Figure 3(d)). These results suggested that sitogluside inhibited melanogenesis *in vitro* and *in vivo*.

### 3.6. Effects of Sitogluside on TYR Expression

**Figure 4:**
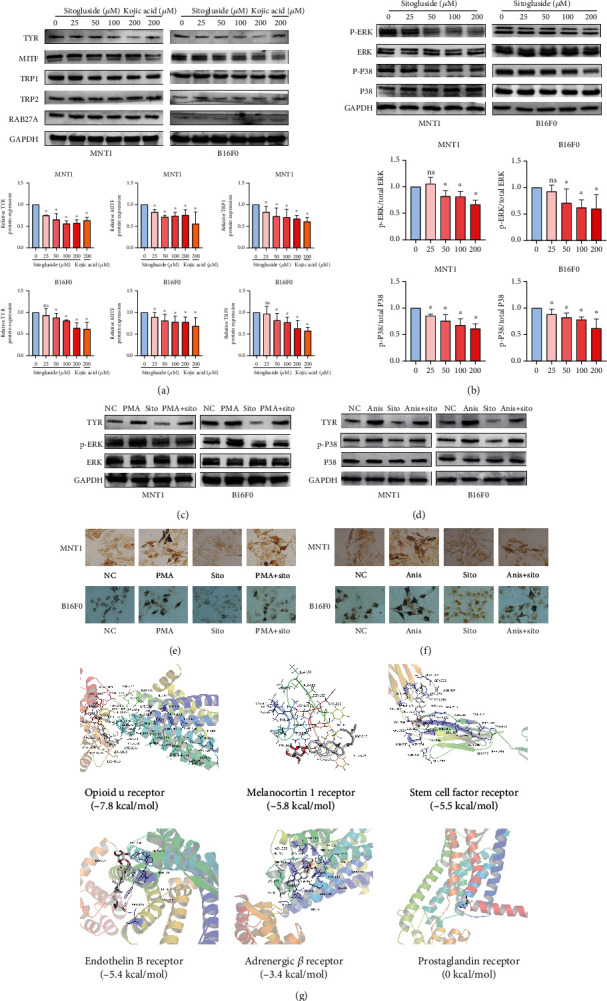
Sitogluside can downregulate the TYR expression through inhibiting the ERK/MAPK and p38/MAPK pathway. (a) The changes of melanogenesis-related gene protein levels in MNT1 and B16F0 cells treated with different concentrations of sitogluside were detected by western blotting. (b) The expression of key proteins in MAPK and PKA pathways was determined by western blotting. (c) Sitogluside can reduce the protein content of TYR by inhibiting the phosphorylation level of ERK activated by PMA. (d) Sitogluside can reduce the protein content of TYR by inhibiting the phosphorylation level of ERK activated by anisomycin. (e) Fontana-Masson melanin staining suggests that sitogluside (200 *μ*M) can reduce melanin content increased by PMA (25 ng/mL). (f) Fontana-Masson melanin staining suggests that sitogluside (200 *μ*M) can reduce melanin content increased by anisomycin (4 nM). (g) The molecular docking between sitogluside and opioid u receptor, melanocortin 1 receptor, stem cell factor receptor, endothelin B receptors, adrenergic *β* receptor, and prostaglandin receptor. PMA: 12-O-tetradecanoyl phorbol-13-acetate; Sito: sitogluside; Anis: anisomycin.

From the previous results, sitogluside can effectively reduce melanin content, but how it regulates this process is still unknown and therefore of interest. Western blotting was performed to detect the expression of melanogenesis-related genes (TYR, MITF, TRP1, TRP2, and RAB27A) at the protein level. The results of the analyses demonstrated that TYR expression was significantly downregulated and MITF and TRP1 expressions were moderately downregulated after sitogluside treatment (Figure 4(a)). Since MAPK and PKA are the key upstream signaling pathways regulating TYR, we further explored the changes in key proteins in these two pathways. After MNT1 and B16F0 cells were treated with sitogluside for 2 days, the phosphorylation levels of ERK and P38 were significantly downregulated, whereas there were no significant changes in the total levels of CREB, ERK, JNK, and p38 proteins or the phosphorylation levels of JNK and CREB (Figure 4(b), Figure S1A). According to the results of CCK8 experiment, we found that PMA below 100 ng/mL is not toxic to both cells (Figure S1B), and 25 ng/mL PMA can obviously increase the phosphorylation level of ERK. The phosphorylation level of ERK gradually decreased after 4 hours (Figure S1C-D). Through Fontana-Masson melanin staining, we found that PMA can significantly increase the melanin content of MNT1 and B16F0 cells. The western blotting results also suggest that PMA can upregulate the phosphorylation level of ERK, and sitogluside can reduce the phosphorylation level of ERK upregulated by PMA. By inhibiting the phosphorylation level of ERK and reducing the protein level of TYR, sitogluside reduces the melanin content increased by PMA (Figures 4(c) and 4(e), Figure S2A). At the same time, we found that anisomycin has similar results. Anisomycin below 8 nM is not toxic to both cells, and 2 nM anisomycin can significantly increase the phosphorylation level of p38. At 8 hours, the phosphorylation level of p38 is still high (Figure S1B, Figure S1E-F). The results of Fontana-Masson melanin staining and western blotting also suggest that sitogluside can inhibit the phosphorylation level of p38 upregulated by anisomycin, reduce the content of TYR protein, and inhibit pigmentation (Figures 4(d) and 4(f), Figure S2B). Therefore, sitogluside may reduce TYR expression by inhibiting the ERK/MAPK and p38/MAPK pathways. However, the mechanism by which sitogluside inhibits these signaling pathways remains unclear. We traced the upstream membrane receptors of these signaling pathways and used molecular docking to explore the mechanism of regulating downstream pathways. The binding energies between sitogluside and the six membrane receptors, viz. the opioid u, melanocortin 1, stem cell factor, endothelin B, adrenergic *β*, and prostaglandin receptors were -7.8, -5.8, -5.5, -5.4, -3.4, and 0 kal/mol, respectively. Through molecular docking, we found that among the six membrane receptors, sitogluside had very high affinities with the opioid u, melanocortin 1, and stem cell growth factor receptors (Figure 4(g)). We speculate that sitogluside may inhibit the ERK/MAPK and p38/MAPK pathways by binding to the before-mentioned membrane receptors, thereby downregulating TYR expression.

### 3.7. Effect of Sitogluside on TYR Activity

**Figure 5:**
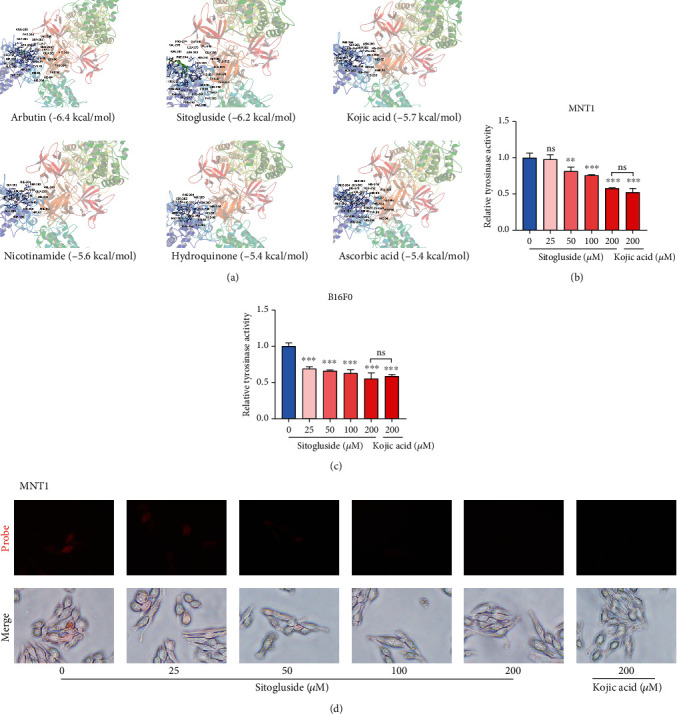
Sitogluside inhibits TYR activity. (a) The molecular docking between sitogluside, arbutin, nicotinamide, hydroquinone, kojic acid, azelaic acid, ascorbic acid, and tyrosinase. (b, c) The tyrosinase activity assay detects the TYR activity of MNT1 and B16F0 after treatment with different concentrations of sitogluside. (d) A near-infrared fluorescence probe was used to detect the TYR activity in MNT1.

Since TYR is the key enzyme in melanin synthesis, it is the most prominent target for inhibiting melanogenesis. Most commercially available cosmetics or skin lightening agents, such as HQ, arbutin, and kojic acid, are TYR inhibitors. We used sitogluside and some clinically used skin-whitening agents (arbutin, nicotinamide, kojic acid, HQ, and ascorbic acid) to conduct molecular docking with TYR. The detailed interactions between sitogluside and the above-mentioned agents with TYR are shown in Figure 5(a). The binding energies of these six compounds were -6.2, -6.4, -5.6, -5.7, -5.4, and -5.4 kcal/mol, respectively. It is generally believed that when the affinity energy is -4, it indicates a binding force, and when it is ≥-7.0, it indicates a strong binding force. Sitogluside ranked second in affinity, slightly weaker than arbutin, exhibiting a relatively outstanding combination ability.

At the same time, L-DOPA conversion experiment was used to detect the activity of TYR, using kojic acid as a positive control. The results showed that sitogluside inhibited TYR activity in MNT1 and B16F0 cells in a concentration-dependent manner, and the high concentration group (200 *μ*M) showed an equivalent inhibitory effect to that shown by 200 *μ*M kojic acid (Figures 5(b) and 5(c)). In addition, the results of TYR probe experiment showed that as the concentration of sitogluside increased, fluorescence intensity gradually decreased in cells, and the fluorescence intensity of the high concentration group was close to that of 200 *μ*M kojic acid in MNT1 and B16F0 cells (Figure 5(d), Figure S2C). These results indicate that sitogluside can effectively inhibit TYR activity in MNT1 and B16F0 cells. Moreover, we speculate that sitogluside, as a natural sterol compound, may directly penetrate the cell membrane and bind to TYR and inhibit its activity, thereby inhibiting melanogenesis.

## 4. Discussion

In this study, we used network pharmacology to explore the mechanisms of potential skin-whitening ingredients found in 14 TCMs, documented in the *Ben-Cao-Gang-Mu* and in folk prescriptions, in inhibiting pigmentation. The study also proposed an innovative method that uses the average “degree” topological parameter of each active ingredient to evaluate the potential effectiveness of the herbs. According to the average “degree,” *Fructus Ligustri Lucidi*, *Hedysarum multijugum Maxim.*, *Ampelopsis japonica*, *Pseudobulbus Cremastrae Seu Pleiones*, and *Paeoniae Radix Alba* were selected, and through the KEGG enrichment analysis of the genes regulated by these five TCMs, it was unexpected that 25% of the enriched signaling pathways were related to inflammation, including IL-17, TNF, HIF-1, PI3K-Akt, and MAPK signaling pathways.

Recently, a variety of inflammatory mediators have been shown to activate or inhibit melanogenesis-related signaling pathways that promote or inhibit skin pigmentation [6]. In cultured B16F10 melanoma cells, TNF-*α* reduced the expression of TYR and TRP1 and TYR activity in a dose-dependent manner [44]. IL-17 and TNF-*α* can reduce the expression of melanogenesis-related genes in melanocytes, and IL-17 can significantly enhance the inhibitory effect of TNF-*α* on melanogenesis. After treatment with etanercept (anti-TNF), melanogenesis-related genes were significantly upregulated in the skin lesions of patients with psoriasis [45]. HIF-1, a powerful transcription factor regulating the inflammatory response, has been reported to be involved in the regulation of factors such as IL-17 and TNF-*α* [46–48]. Therefore, HIF-1 is likely to participate in the regulation of melanogenesis by influencing inflammation. Other cytokines such as IL-18, IL-33, GM-CSF, and IL-1*α* can promote melanogenesis by activating the PKA, MAPK, or other signaling pathways, while IFN-*γ*, IL-1*β*, IL-4, and IL-6 reduce melanogenesis by inhibiting STAT1, NF-*κ*B, and JAK2-STAT6 signaling pathways [6]. Our findings suggest that the top five TCMs (*Fructus Ligustri Lucidi*, *Hedysarum multijugum Maxim.*, *Ampelopsis japonica*, *Pseudobulbus Cremastrae Seu Pleiones*, and *Paeoniae Radix Alba*) may exert an antipigmentation effect mainly through anti-inflammatory pathways. Previous literature also mentioned that these top five TCMs have powerful anti-inflammatory and antiaging effects [49, 50]. This also supports our conjecture that TCMs may play a nonnegligible role in postinflammatory pigmentation.

While perusing the literature of 148 ingredients found in 14 TCMs, we also found that among 35 of the reported ingredients, nearly 70% of them were confirmed to either reduce melanin content or inhibit TYR activity. This not only confirms that records of skin-whitening prescriptions in ancient books are largely reliable but also shows that reference to ancient literature is a dependable method to obtain knowledge of TCMs. There are still 95 compounds that remain unexplored, which have the potential to resist pigmentation. Through analysis of the Venn diagrams of the top five TCMs, 13 common ingredients were obtained. Among them, *in vitro* experiments confirmed that daidzein, rutin, and (+)-catechin can reduce melanin content [51–53]. Sucrose disturbs proper melanosome maturation by inducing osmotic stress and inhibiting the PI3 kinase pathway, thereby interfering with melanogenesis [54]. Quercetin reduces oxidative stress-induced melanogenesis [55]. In addition, chemical and physical methods such as spectroscopic and structure-activity analyses confirmed that lupeol and kaempferol can inhibit TYR and reduce melanogenesis [56, 57]. This indicates that network pharmacology maybe is a feasible method to dig out whitening ingredients. Four TCMs in the top five TCMs contained sitogluside, and there are no reports on whether sitogluside regulates pigmentation or its related mechanism. Therefore, we chose sitogluside for these studies.

Sitogluside, a sterol glycoside that is widely present in plants, has been reported to exert antitumor, antioxidative, and neuroprotective effects [58–60]. In this study, we discovered that sitogluside effectively reduced melanin content *in vitro* and *in vivo*. Subsequent experiments proved that it could significantly reduce the expression of TYR. Kojic acid was used as a positive control, and it can also inhibit the protein levels of melanogenesis-related genes such as TYR, MITF, and TYRP1 under *α*-MSH stimulation, which was reported by Jeon et al. [61]. Given the absence of exogenous stimulation, 200 *μ*M kojic acid had only moderate inhibitory effects on gene expression in our study, but because kojic acid has great tyrosinase inhibitory activity, we still use it as a positive control in this study. TYR expression was reported to be directly or indirectly regulated by the MAPK or PKA pathways, respectively. An increasing number of ingredients have been confirmed to have a whitening effect on the skin by inhibiting the MAPK signaling pathway. For example, fargesin reduces the expression of TYR by inhibiting the P38/MAPK pathway [62]. Ganoderma lucidum polysaccharides can resist UVB-induced melanogenesis by downregulating the MAPK pathway [63]. Our study found that sitogluside, at a concentration of 200 *μ*M, had the potential to reduce TYR expression by inhibiting ERK/MAPK and P38/MAPK signaling pathways. This has been reported through the molecular docking between the upstream membrane receptors of MAPK pathways [64] and sitogluside. We found that sitogluside binds strongly to the opioid u, melanocortin 1, and stem cell growth factor receptors. Therefore, we speculate that sitogluside may interact with these membrane receptors, inhibit the ERK/MAPK and P38/MAPK signaling pathways, and reduce the expression of TYR.

**Figure 6:**
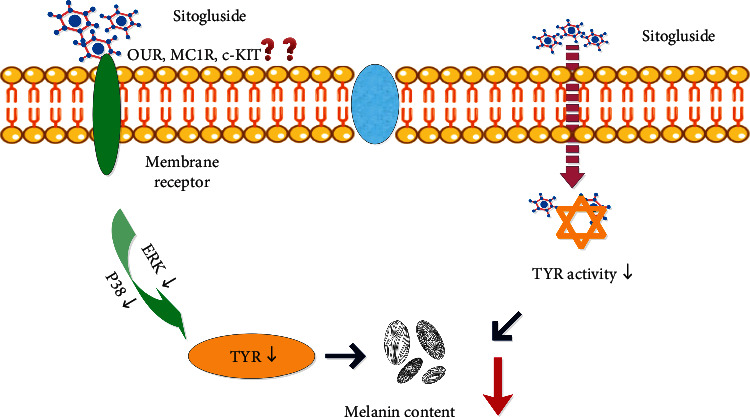
Sitogluside, the most common ingredient in the top five TCMs selected by network pharmacology, as small molecular compound of sterol, on the one hand, may reduce the expression of TYR by inhibiting the ERK and p38 pathway through binding to membrane receptors (such as OUR, MC1R, and C-KIT). On the other hand, it may penetrate the cell membrane, directly bind to tyrosinase, inhibit the activity of TYR, and reduce the melanin content. c-KIT: stem cell growth factor receptor; OUR: opioid u receptor; MC1R: melanocortin 1 receptor; TCM: traditional Chinese medicine; TYR: tyrosinase.

Since TYR is the key enzyme in the synthesis of melanin particles in melanocytes, inhibiting the activity of the catalytic site of TYR is essential to reduce skin pigmentation [65]. Many skin-lightening agents in the market are TYR activity inhibitors, such as HQ, arbutin, and kojic acid. In this study, we used molecular docking to explore the binding effects of sitogluside and five common skin-whitening active ingredients (arbutin, nicotinamide, kojic acid, HQ, and ascorbic acid) [66] with TYR. The results showed that sitogluside had a strong binding affinity with TYR, weaker than arbutin, but stronger than kojic acid, nicotinamide, HQ, and ascorbic acid. Arbutin is a prodrug of HQ and a natural product that can reduce or inhibit the synthesis of melanin by inhibiting TYR. However, the natural form of arbutin is chemically unstable and can convert to HQ, which is catalyzed and metabolized into benzene metabolites with potential toxicity to bone marrow [67]. The use of kojic acid in cosmetics is restricted because of its carcinogenicity and instability during storage [68]. Therefore, sitogluside, a natural sterol compound derived from a variety of plants, is expected to act as a potent skin-lightening agent. However, the solubility of sitogluside in DMSO is low, which is also a shortcoming of this natural ingredient. Subsequent experimental verification revealed that sitogluside is safe in MNT1 and B16F0 cell lines, and the low concentration group had a good inhibitory effect on TYR activity, while the high concentration group had the same effect as the same concentration of kojic acid. Therefore, sitogluside may be therapeutically used as a skin-whitening regulator, which not only reduces the expression of TYR but also inhibits its activity (Figure 6).

## 5. Conclusions

The network pharmacology is maybe an effective tool for the discovery of natural skin-pigmentation-reducing active ingredients and their possible mechanisms. *Fructus Ligustri Lucidi*, *Hedysarum multijugum maxim.*, *Ampelopsis japonica*, *Pseudobulbus Cremastrae Seu Pleiones*, and *Paeoniae Radix Alba* have potential skin-whitening properties and may inhibit pigmentation by regulating inflammation. Sitogluside is a novel skin-whitening active ingredient with dual regulatory effects, viz. inhibiting TYR expression and activity.

## Data Availability

The data used to support the findings of this study are available from the corresponding author upon request.

## Supplementary Materials

Supplementary Materials Figure S1: (A) expression of p-CREB, CREB, p-JUK, and JUK in MAPK and PKA pathways was determined by western blotting. (B) CCK8 assay detects the cell viability of MNT1 and B16F0 cells treated with different concentrations of PMA and anisomycin, respectively. (C) PMA can significantly upregulate the phosphorylation level of ERK in MNT1 and B16F0 cells. (D) Within eight hours, PMA significantly increased the phosphorylation level of ERK. (E) Anisomycin can significantly upregulate the phosphorylation level of p38 in MNT1 and B16F0 cells. (F) Within eight hours, anisomycin significantly increased the phosphorylation level of p38. Figure S2: (A, B) The statistical results of western blotting of function recovery experiment in MNT1 and B16F0. (C) A near-infrared fluorescence probe was used to detect the TYR activity in B16F0. Table S1: basic information of traditional Chinese medicine in Ben-Cao-Gang-Mu and folk prescriptions. Table S2: the Venn analysis of potential effective components of 5 traditional Chinese medicines. Table S3: details of the potential active ingredients of 14 traditional Chinese medicines.

## References

[1] Pillaiyar T., Namasivayam V., Manickam M., Jung S. H. (2018). Inhibitors of melanogenesis: an updated review. *Journal of Medicinal Chemistry*.

[2] Ito S. (2003). A Chemist's View of Melanogenesis. *Pigment Cell Research*.

[3] Yuan X. H., Jin Z. H. (2018). Paracrine regulation of melanogenesis. *The British Journal of Dermatology*.

[4] Lu D., Lin X., Chen C. (2020). Interference-free SERS tags for ultrasensitive quantitative detection of tyrosinase in human serum based on magnetic bead separation. *Analytica Chimica Acta*.

[5] Park H. Y., Wu C., Yonemoto L. (2006). MITF mediates cAMP-induced protein kinase C-*β* expression in human melanocytes. *The Biochemical Journal*.

[6] Fu C., Chen J., Lu J. (2020). Roles of inflammation factors in melanogenesis (review). *Molecular Medicine Reports*.

[7] Zhou J., Shang J., Song J., Ping F. (2013). Interleukin-18 augments growth ability of primary human melanocytes by PTEN inactivation through the AKT/NF-*κ*B pathway. *The International Journal of Biochemistry & Cell Biology*.

[8] Yun W., Li C. (2010). JNK pathway is required for TNCB-induced IL-18 expression in murine keratinocytes. *Toxicology In Vitro*.

[9] Englaro W., Bahadoran P., Bertolotto C. (1999). Tumor necrosis factor alpha-mediated inhibition of melanogenesis is dependent on nuclear factor kappa B activation. *Oncogene*.

[10] Scott G., Leopardi S., Printup S., Malhi N., Seiberg M., Lapoint R. (2004). Proteinase-Activated Receptor-2 Stimulates Prostaglandin Production in Keratinocytes: Analysis of Prostaglandin Receptors on Human Melanocytes and Effects of PGE_2_ and PGF_2*α*_ on Melanocyte Dendricity. *The Journal of Investigative Dermatology*.

[11] Kaufman B. P., Aman T., Alexis A. F. (2018). Postinflammatory hyperpigmentation: epidemiology, clinical presentation, pathogenesis and treatment. *American Journal of Clinical Dermatology*.

[12] Agbai O., Hamzavi I., Jagdeo J. (2017). Laser treatments for postinflammatory Hyperpigmentation. *JAMA Dermatology*.

[13] Wang X., Zhang Y. (2018). Resveratrol alleviates LPS-induced injury in human keratinocyte cell line HaCaT by up-regulation of miR-17. *Biochemical and Biophysical Research Communications*.

[14] Eo S. H., Kim S. J. (2019). Resveratrol-mediated inhibition of cyclooxygenase-2 in melanocytes suppresses melanogenesis through extracellular signal-regulated kinase 1/2 and phosphoinositide 3-kinase/Akt signalling. *European Journal of Pharmacology*.

[15] Desmedt B., Courselle P., De Beer J. O. (2016). Overview of skin whitening agents with an insight into the illegal cosmetic market in Europe. *Journal of the European Academy of Dermatology and Venereology*.

[16] Nordlund J. J., Grimes P. E., Ortonne J. P. (2006). The safety of hydroquinone. *Journal of the European Academy of Dermatology and Venereology*.

[17] Karamagi C., Owino E., Katabira E. T. (2001). Hydroquinone neuropathy following use of skin bleaching cream: case report. *East African Medical Journal*.

[18] Al-Niaimi F., Chiang N. Y. Z. (2017). Topical vitamin C and the skin: mechanisms of action and clinical applications. *The Journal of Clinical and Aesthetic Dermatology*.

[19] Hu Y., Zeng H., Huang J., Jiang L., Chen J., Zeng Q. (2020). Traditional Asian herbs in skin whitening: the current development and limitations. *Frontiers in Pharmacology*.

[20] Yoshihisa Y., Andoh T., Rehman M. U., Shimizu T. (2020). The regulation of protein kinase casein kinase II by apigenin is involved in the inhibition of ultraviolet B-induced macrophage migration inhibitory factor-mediated hyperpigmentation. *Phytotherapy Research*.

[21] Qiu J., Chen M., Liu J. (2016). The skin-depigmenting potential of Paeonia lactiflora root extract and paeoniflorin:in vitroevaluation using reconstructed pigmented human epidermis. *International Journal of Cosmetic Science*.

[22] Tsao Y. T., Kuo C. Y., Kuan Y. D., Lin H. C., Wu L. H., Lee C. H. (2017). The extracts ofAstragalus membranaceusInhibit melanogenesis through the ERK signaling pathway. *International Journal of Medical Sciences*.

[23] Gong S., Yang Z. (2004). Microwave-assisted extraction technology of skin-lightning agent in Rhizoma Typhonii. *Zhong Yao Cai*.

[24] Wei M. P., Qiu J. D., Li L. (2021). Saponin fraction from _Sapindus mukorossi_ Gaertn as a novel cosmetic additive: Extraction, biological evaluation, analysis of anti-acne mechanism and toxicity prediction. *Journal of Ethnopharmacology*.

[25] Ye Y., Chu J. H., Wang H. (2010). Involvement of p38 MAPK signaling pathway in the anti-melanogenic effect of san-bai-tang, a Chinese herbal formula, in B16 cells. *Journal of Ethnopharmacology*.

[26] Huang H. C., Hsieh W. Y., Niu Y. L., Chang T. M. (2014). Inhibitory effects of adlay extract on melanin production and cellular oxygen stress in B16F10 melanoma cells. *International Journal of Molecular Sciences*.

[27] Ye Y., Chou G. X., Mu D. D. (2010). Screening of Chinese herbal medicines for antityrosinase activity in a cell free system and B16 cells. *Journal of Ethnopharmacology*.

[28] Li Y., Huang J., Lu J. (2019). The role and mechanism of Asian medicinal plants in treating skin pigmentary disorders. *Journal of Ethnopharmacology*.

[29] Poornima P., Kumar J. D., Zhao Q., Blunder M., Efferth T. (2016). Network pharmacology of cancer: from understanding of complex interactomes to the design of multi-target specific therapeutics from nature. *Pharmacological Research*.

[30] Li S., Zhang B. (2013). Traditional Chinese medicine network pharmacology: theory, methodology and application. *Chinese Journal of Natural Medicines*.

[31] Li P., Chen J., Wang J. (2014). Systems pharmacology strategies for drug discovery and combination with applications to cardiovascular diseases. *Journal of Ethnopharmacology*.

[32] Meng Z. Q., Wu J. R., Zhu Y. L. (2021). Revealing the common mechanisms of scutellarin in angina pectoris and ischemic stroke treatment via a network pharmacology approach. *Chinese Journal of Integrative Medicine*.

[33] Ru J., Li P., Wang J. (2014). TCMSP: a database of systems pharmacology for drug discovery from herbal medicines. *Journal of Cheminformatics*.

[34] Ritchie M. E., Phipson B., Wu D. (2015). limma powers differential expression analyses for RNA-sequencing and microarray studies. *Nucleic acids research*.

[35] Franz M., Lopes C. T., Huck G., Dong Y., Sumer O., Bader G. D. (2015). Cytoscape.js: a graph theory library for visualisation and analysis. *Bioinformatics*.

[36] Zhao Z., Liu G., Meng Y. (2019). Synthesis and anti-tyrosinase mechanism of the substituted vanillyl cinnamate analogues. *Bioorganic Chemistry*.

[37] Trott O., Olson A. J. (2009). AutoDock Vina: improving the speed and accuracy of docking with a new scoring function, efficient optimization, and multithreading. *Journal of Computational Chemistry*.

[38] Benito-Martinez S., Zhu Y., Jani R. A., Harper D. C., Marks M. S., Delevoye C. (2020). Research techniques made simple: cell biology methods for the analysis of pigmentation. *The Journal of Investigative Dermatology*.

[39] Hiratsuka T., Fujita Y., Naoki H., Aoki K., Kamioka Y., Matsuda M. (2015). Intercellular propagation of extracellular signal-regulated kinase activation revealed by in vivo imaging of mouse skin. *eLife*.

[40] Refsnes M., Skuland T., Schwarze P., Lag M., Ovrevik J. (2014). Differential NF*κ*B and MAPK activation underlies fluoride- and TPA-mediated CXCL8 (IL-8) induction in lung epithelial cells. *Journal of Inflammation Research*.

[41] Mawji I. A., Simpson C. D., Gronda M. (2007). A chemical screen identifies anisomycin as an anoikis sensitizer that functions by decreasing FLIP protein synthesis. *Cancer Research*.

[42] Wu X., Li L., Shi W., Gong Q., Ma H. (2016). Near-infrared fluorescent probe with new recognition moiety for specific detection of tyrosinase activity: design, synthesis, and application in living cells and zebrafish. *Angewandte Chemie (International Ed. in English)*.

[43] Ko G. A., Kang H. R., Moon J. Y. (2020). Annona squamosaL. leaves inhibit alpha-melanocyte-stimulating hormone (*α*‐MSH) stimulated melanogenesis via p38 signaling pathway in B16F10 melanoma cells. *Journal of Cosmetic Dermatology*.

[44] Shen X. L., Liu Y. Z., Gong H. (2020). Chronic stress induces fur color change from dark to brown by decreasing follicle melanocytes and tyrosinase activity in female C57BL/6 mice. *Sheng Li Xue Bao*.

[45] Wang C. Q. F., Akalu Y. T., Suarez-Farinas M. (2013). IL-17 and TNF synergistically modulate cytokine expression while suppressing melanogenesis: potential relevance to psoriasis. *The Journal of Investigative Dermatology*.

[46] Zhao W., Wu C., Li L. J. (2018). RNAi silencing of HIF-1*α* ameliorates lupus development in MRL/lpr mice. *Inflammation*.

[47] Dang E. V., Barbi J., Yang H. Y. (2011). Control of T_H_17/T_reg_ Balance by Hypoxia-Inducible Factor 1. *Cell*.

[48] Gao X., Li Y., Wang H., Li C., Ding J. (2017). Inhibition of HIF-1*α* decreases expression of pro-inflammatory IL-6 and TNF-*α* in diabetic retinopathy. *Acta Ophthalmologica*.

[49] Li L., Chen B., Zhu R. (2019). Fructus Ligustri Lucidi preserves bone quality through the regulation of gut microbiota diversity, oxidative stress, TMAO and Sirt6 levels in aging mice. *Aging (Albany NY)*.

[50] Liu P., Zhao H., Luo Y. (2017). Anti-aging implications of Astragalus membranaceus (Huangqi): a well-known Chinese tonic. *Aging and Disease*.

[51] Wang H. Z., Zhang Y., Xie L. P., Yu X. Y., Zhang R. Q. (2002). Effects of genistein and daidzein on the cell growth, cell cycle, and differentiation of human and murine melanoma cells^1^. *The Journal of Nutritional Biochemistry*.

[52] Drewa G., Schachtschabel D. O., Palgan K., Grzanka A., Sujkowska R. (1998). The influence of rutin on the weight, metastasis and melanin content of B16 melanotic melanoma in C57BL/6 mice. *Neoplasma*.

[53] Fujimaki T., Mori S., Horikawa M., Fukui Y. (2018). Isolation of proanthocyanidins from red wine, and their inhibitory effects on melanin synthesis _in vitro_. *Food Chemistry*.

[54] Bin B. H., Kim S. T., Bhin J., Lee T. R., Cho E. G. (2016). The development of sugar-based anti-melanogenic agents. *International Journal of Molecular Sciences*.

[55] Kim Y. J. (2012). Hyperin and quercetin modulate oxidative stress-induced melanogenesis. *Biological & Pharmaceutical Bulletin*.

[56] Myint K. Z. W., Kido T., Kusakari K., Devkota H. P., Kawahara T., Watanabe T. (2019). Rhusflavanone and mesuaferrone B: tyrosinase and elastase inhibitory biflavonoids extracted from the stamens of *Mesua ferrea* L. *Natural Product Research*.

[57] Rho H. S., Ghimeray A. K., Yoo D. S. (2011). Kaempferol and kaempferol rhamnosides with depigmenting and anti-inflammatory properties. *Molecules*.

[58] Zeng J., Liu X., Li X., Zheng Y., Liu B., Xiao Y. (2017). Daucosterol inhibits the proliferation, migration, and invasion of hepatocellular carcinoma cells via Wnt/*β*-Catenin signaling. *Molecules*.

[59] Osman S. M., El-Haddad A. E., El-Raey M. A., Abd El-Khalik S. M., Koheil M. A., Wink M. (2016). A new octadecenoic acid derivative from Caesalpinia gilliesii flowers with potent hepatoprotective activity. *Pharmacognosy Magazine*.

[60] Jiang L. H., Yuan X. L., Yang N. Y. (2015). Daucosterol protects neurons against oxygen-glucose deprivation/reperfusion- mediated injury by activating IGF1 signaling pathway. *The Journal of Steroid Biochemistry and Molecular Biology*.

[61] Jeon H. J., Kim K., Kim C., Kim M. J., Kim T. O., Lee S. E. (2021). Molecular mechanisms of anti-melanogenic gedunin derived from neem tree (Azadirachta indica) using B16F10 mouse melanoma cells and early-stage zebrafish. *Plants*.

[62] Fu T., Chai B., Shi Y., Dang Y., Ye X. (2019). Fargesin inhibits melanin synthesis in murine malignant and immortalized melanocytes by regulating PKA/CREB and P38/MAPK signaling pathways. *Journal of Dermatological Science*.

[63] Hu S., Huang J., Pei S. (2019). Ganoderma lucidumpolysaccharide inhibits UVB-induced melanogenesis by antagonizing cAMP/PKA and ROS/MAPK signaling pathways. *Journal of Cellular Physiology*.

[64] Slominski A., Tobin D. J., Shibahara S., Wortsman J. (2004). Melanin pigmentation in mammalian skin and its hormonal regulation. *Physiological Reviews*.

[65] Bijelic A., Pretzler M., Molitor C., Zekiri F., Rompel A. (2015). The structure of a plant tyrosinase from walnut leaves reveals the importance of “substrate-guiding residues” for enzymatic specificity. *Angewandte Chemie (International Ed. in English)*.

[66] Pillaiyar T., Manickam M., Namasivayam V. (2017). Skin whitening agents: medicinal chemistry perspective of tyrosinase inhibitors. *Journal of Enzyme Inhibition and Medicinal Chemistry*.

[67] Zhou H., Kepa J. K., Siegel D., Miura S., Hiraki Y., Ross D. (2009). Benzene metabolite hydroquinone up-regulates chondromodulin-I and inhibits tube formation in human bone marrow endothelial cells. *Molecular Pharmacology*.

[68] Karami S., Boffetta P., Rothman N. (2008). Renal cell carcinoma, occupational pesticide exposure and modification by glutathione S-transferase polymorphisms. *Carcinogenesis*.

