# Supplementary material for: Identification of Sitogluside as a Potential Skin-Pigmentation-Reducing Agent through Network Pharmacology
Source: Oxid Med Cell Longev. 2021 Sep 23;2021:4883398. doi: 10.1155/2021/4883398 (PMC8483913; doi:10.1155/2021/4883398)
Supplement: Supplementary Materials — Figure S1: (A) expression of p-CREB, CREB, p-JUK, and JUK in MAPK and PKA pathways was determined by western blotting. (B) CCK8 assay detects the cell viability of MNT1 and B16F0 cells treated with different concentrations of PMA and anisomycin, respectively. (C) PMA can significantly upregulate the phosphorylation level of ERK in MNT1 and B16F0 cells. (D) Within eight hours, PMA significantly increased the phosphorylation level of ERK. (E) Anisomycin can significantly upregulate the phosphorylation level of p38 in MNT1 and B16F0 cells. (F) Within eight hours, anisomycin significantly increased the phosphorylation level of p38. Figure S2: (A, B) The statistical results of western blotting of function recovery experiment in MNT1 and B16F0. (C) A near-infrared fluorescence probe was used to detect the TYR activity in B16F0. Table S1: basic information of traditional Chinese medicine in Ben-Cao-Gang-Mu and folk prescriptions. Table S2: the Venn analysis of potential effective components of 5 traditional Chinese medicines. Table S3: details of the potential active ingredients of 14 traditional Chinese medicines. [file 4883398.f1.zip › supplementary Table (1).docx]

**Table S1：Basic information of traditional Chinese medicine in *Ben-Cao-Gang-Mu* and folk prescriptions**

|  |  | **Traditional Chinese medicine** | **Number of Ingredients (DL>0.18)** |
| --- | --- | --- | --- |
| ***Ben-Cao-Gang-Mu*** |  | *Typhonii Rhizoma* | 7 |
|  |  | *Ricini Semen* | 6 |
|  |  | *Hedysarum Multijugum Maxim.* | 47 |
|  |  | *Pseudobulbus Cremastrae Seu Pleiones* | 12 |
|  |  | *Sapindi Mukorossi Semen* | 11 |
| **Folk prescriptions** | *San-Bai-Tang* | *Poria Cocos (Schw.) Wolf.* | 25 |
|  | *San-Bai-Tang* | *Paeoniae Radix Alba* | 37 |
|  | *San-Bai-Tang* | *Atractylodes Macrocephala Koidz.* | 10 |
|  |  | *Blethun Striata (Bletilla Striata. Murray) Rchb.F.* | 28 |
|  |  | *Ampelopsis Japonica* | 26 |
|  |  | *A. Dahurica (Fisch.) Benth. Et Hook* | 45 |
|  |  | *Atractylodes Lancea (Thunb.) Dc.* | 20 |
|  |  | *Fructus Ligustri Lucidi* | 80 |
|  |  | *Coicis Semen* | 18 |

**DL:Druglike**

**Table S2：The Venn Analysis of Potential Effective Components of 5 Traditional Chinese medicines**

| **Traditional Chinese medicines** | **Co-ingredient** |
| --- | --- |
| AJ FLL PCSP PRA | Sitogluside, beta-sitosterol |
| AJ FLL HMM | quercetin |
| FLL HMM PRA | kaempferol |
| AJ FLL PRA | oleanolic acid |
| FLL HMM | Daidzein, rutin |
| FLL PRA | sucrose |
| AJ HMM | lupeol |
| HMM PRA | Mairin |
| AJ PCSP | Stigmasterol |
| AJ PRA | (+)-catechin, sitosterol |

AJ: *[Ampelopsis Japonica](http://tcmspw.com/tcmspsearch.php?qr=Ampelopsis%20Japonica&qsr=herb_en_name&token=fb321389429665aba69eb20f7267aa39)*; HMM: *[Hedysarum Multijugum Maxim.](http://tcmspw.com/tcmspsearch.php?qr=Hedysarum%20Multijugum%20Maxim.&qsr=herb_en_name&token=fb321389429665aba69eb20f7267aa39)*; FLL: *[Fructus Ligustri Lucidi](http://tcmspw.com/tcmspsearch.php?qr=Fructus%20Ligustri%20Lucidi&qsr=herb_en_name&token=fb321389429665aba69eb20f7267aa39)*; PCSP: *Pseudobulbus Cremastrae Seu Pleiones*; PRA: *[Paeoniae Radix Alba](http://tcmspw.com/tcmspsearch.php?qr=Paeoniae%20Radix%20Alba&qsr=herb_en_name&token=fb321389429665aba69eb20f7267aa39)*.

**Table S3：Details of the potential active ingredients of 14 Traditional Chinese Medicines**

| **Ingredient** | **Traditional Chinese medicine** | **Reported to be related to melanin** | | | **Effect on melanogenesis** | **references** |
| --- | --- | --- | --- | --- | --- | --- |
|  |  | **Reported** | **extract** | **derivative** |  |  |
| Sitogluside/daucosterol | Typhonii Rhizoma，Pseudobulbus Cremastrae Seu Pleiones，Paeoniae Radix Alba，Ampelopsis Japonica，A. Dahurica (Fisch.) Benth. Et Hook，Fructus Ligustri Lucidi,  Atractylodes Lancea (Thunb.) Dc. | yes |  |  | unclear | PMID:22468750 |
| Beta-sitosterol | Typhonii Rhizoma，Pseudobulbus Cremastrae Seu Pleiones，Paeoniae Radix Alba，Ampelopsis Japonica，A. Dahurica (Fisch.) Benth. Et Hook，Fructus Ligustri Lucidi |  | A. squamosa leaf extract (ent-kaur-16-en-19-ol, 18-oxokauran-17-yl acetate, and β-sitosterol) |  | inhibitition | PMID:31763737 |
| Sitosterol | Typhonii Rhizoma，Paeoniae Radix Alba，Ampelopsis Japonica，Coicis Semen |  |  | beta-sitosterol | inhibitition | PMID:31763737 |
| Stigmasterol | Pseudobulbus Cremastrae Seu Pleiones， Ampelopsis Japonica，A. Dahurica (Fisch.) Benth. Et Hook，Coicis Semen | YES |  |  | unclear | PMID: 23891889 |
| Kaempferol | Hedysarum Multijugum Maxim.，Paeoniae Radix Alba，Fructus Ligustri Lucidi | YES |  |  | inhibitition | PMID:21512441 |
| Quercetin | Hedysarum Multijugum Maxim.，Ampelopsis Japonica，Fructus Ligustri Lucidi | YES |  |  | inhibitition | PMID:22971648 |
| Oleanolic acid | Paeoniae Radix Alba，Ampelopsis Japonica， Fructus Ligustri Lucidi |  |  | oleanolic acid 3-O-β-D-glucopyranosyl (1→3)-β-D-glucopyranosiduronic acid | Promotion | PMID;26887328 |
| Sucrose | Paeoniae Radix Alba，A. Dahurica (Fisch.) Benth. Et Hook， Fructus Ligustri Lucidi | YES |  |  | inhibitition | PMID:27092497 |
| Mairin | Hedysarum Multijugum Maxim.，Paeoniae Radix Alba | NO |  |  |  |  |
| Alexandrin | Hedysarum Multijugum Maxim.，Poria Cocos (Schw.) Wolf. | NO |  |  |  |  |
| Hederagenin | Hedysarum Multijugum Maxim.，Poria Cocos (Schw.) Wolf. | NO |  |  |  |  |
| (3S,8S,9S,10R,13R,14S,17R)-10,13-dimethyl-17-[(2R,5S)-5-propan-2-yloctan-2-yl]-2,3,4,7,8,9,11,12,14,15,16,17-dodecahydro-1H-cyclopenta[a]phenanthren-3-ol | Hedysarum Multijugum Maxim.，A. Dahurica (Fisch.) Benth. Et Hook | NO |  |  |  |  |
| Lupeol | Hedysarum Multijugum Maxim.，Ampelopsis Japonica | YES |  |  | inhibitition | PMID:31135222 |
| Daidzein | Hedysarum Multijugum Maxim.， Fructus Ligustri Lucidi | YES |  |  | inhibitition | PMID:12121829 |
| Rutin | Hedysarum Multijugum Maxim.， Fructus Ligustri Lucidi | YES |  |  | inhibitition | PMID:9890672 |
| Ergosterol | Poria Cocos (Schw.) Wolf.，Coicis Semen |  |  | ergosterol peroxide | inhibitition | PMID:20134236 |
| (+)-catechin | Paeoniae Radix Alba，Ampelopsis Japonica | YES |  |  | inhibitition | PMID:29329871 |
| 3β-acetoxyatractylone | Atractylodes Macrocephala Koidz.，Atractylodes Lancea (Thunb.) Dc. | NO |  |  |  |  |
| Physcion | Bletilla Striata (Thunb.Ex A.Murray)Rchb.F.，Ampelopsis Japonica | YES |  |  | inhibitition | PMID:18338768 |
| Mandenol | A. Dahurica (Fisch.) Benth. Et Hook，Coicis Semen | NO |  |  |  |  |
| CLR | A. Dahurica (Fisch.) Benth. Et Hook，Coicis Semen | NO |  |  |  |  |
| Mandenol | Typhonii Rhizoma | NO |  |  |  |  |
| 7beta,12-Dimethoxy-8,11,13-abietatrien-11-ol | Ricini Semen | NO |  |  |  |  |
| Costaclavine | Ricini Semen | NO |  |  |  |  |
| Riddelline | Ricini Semen | NO |  |  |  |  |
| Jaranol | Hedysarum Multijugum Maxim. | YES |  |  | inhibitition | PMID:27746860 |
| Rhamnocitrin | Hedysarum Multijugum Maxim. | NO |  |  |  |  |
| Isorhamnetin | Hedysarum Multijugum Maxim. | YES |  |  | Promotion | PMID:29145845 |
| 3,9-di-O-methylnissolin | Hedysarum Multijugum Maxim. | NO |  |  |  |  |
| (2S)-4-methoxy-7-methyl-2-[1-methyl-1-[(2S,3R,4S,5S,6R)-3,4,5-trihydroxy-6-methylol-tetrahydropyran-2-yl]oxy-ethyl]-2,3-dihydrofuro[3,2-g]chromen-5-one | Hedysarum Multijugum Maxim. | NO |  |  |  |  |
| 7-O-methylisomucronulatol | Hedysarum Multijugum Maxim. | NO |  |  |  |  |
| 9,10-dimethoxypterocarpan-3-O-β-D-glucoside | Hedysarum Multijugum Maxim. | NO |  |  |  |  |
| (6aR,11aR)-9,10-dimethoxy-6a,11a-dihydro-6H-benzofurano[3,2-c] chromen-3-ol | Hedysarum Multijugum Maxim. | NO |  |  |  |  |
| Bifendate | Hedysarum Multijugum Maxim. | NO |  |  |  |  |
| Ononin | Hedysarum Multijugum Maxim. | YES |  |  | unclear | PMID:22738950 |
| Formononetin | Hedysarum Multijugum Maxim. | YES |  |  | inhibitition | PMID:29873272 |
| (+)-Syringaresinol | Hedysarum Multijugum Maxim. |  | ginseng berry extract |  | inhibitition | PMID:28701867 |
| Flavaxin | Hedysarum Multijugum Maxim. | NO |  |  |  |  |
| Astraisoflavanin | Hedysarum Multijugum Maxim. | NO |  |  |  |  |
| Mucronulatol | Hedysarum Multijugum Maxim. | NO |  |  |  |  |
| Lariciresinol | Hedysarum Multijugum Maxim. | NO |  |  |  |  |
| Calycosin | Hedysarum Multijugum Maxim. | YES |  |  | iinhibitition | PMID:19182387 |
| 3'-Hydroxy-4'-methoxyisoflavone-7-O-beta-D-glucoside | Hedysarum Multijugum Maxim. | NO |  |  |  |  |
| FA | Hedysarum Multijugum Maxim. | NO |  |  |  |  |
| 1,7-Dihydroxy-3,9-dimethoxy pterocarpene | Hedysarum Multijugum Maxim. | NO |  |  |  |  |
| Loroglossin_qt | [Pseudobulbus Cremastrae Seu Pleiones](http://tcmspw.com/tcmspsearch.php?qr=Pseudobulbus%20Cremastrae%20Seu%20Pleiones&qsr=herb_en_name&token=fb321389429665aba69eb20f7267aa39) | NO |  |  |  |  |
| Militarin_qt | [Pseudobulbus Cremastrae Seu Pleiones](http://tcmspw.com/tcmspsearch.php?qr=Pseudobulbus%20Cremastrae%20Seu%20Pleiones&qsr=herb_en_name&token=fb321389429665aba69eb20f7267aa39) | NO |  |  |  |  |
| 2-methoxy-9,10-dihydrophenanthrene-4,5-diol | [Pseudobulbus Cremastrae Seu Pleiones](http://tcmspw.com/tcmspsearch.php?qr=Pseudobulbus%20Cremastrae%20Seu%20Pleiones&qsr=herb_en_name&token=fb321389429665aba69eb20f7267aa39) | NO |  |  |  |  |
| Flavanthrinin | [Pseudobulbus Cremastrae Seu Pleiones](http://tcmspw.com/tcmspsearch.php?qr=Pseudobulbus%20Cremastrae%20Seu%20Pleiones&qsr=herb_en_name&token=fb321389429665aba69eb20f7267aa39) | NO |  |  |  |  |
| Arachic acid | [Sapindi Mukorossi Semen](http://tcmspw.com/tcmspsearch.php?qr=Sapindi%20Mukorossi%20Semen&qsr=herb_en_name&token=fb321389429665aba69eb20f7267aa39) | NO |  |  |  |  |
| Mulberrin | [Sapindi Mukorossi Semen](http://tcmspw.com/tcmspsearch.php?qr=Sapindi%20Mukorossi%20Semen&qsr=herb_en_name&token=fb321389429665aba69eb20f7267aa39) | NO |  |  |  |  |
| 1,6-Dihydroxy-3,7-dimethoxyxanthone | [Sapindi Mukorossi Semen](http://tcmspw.com/tcmspsearch.php?qr=Sapindi%20Mukorossi%20Semen&qsr=herb_en_name&token=fb321389429665aba69eb20f7267aa39) | NO |  |  |  |  |
| Erucic acid | [Sapindi Mukorossi Semen](http://tcmspw.com/tcmspsearch.php?qr=Sapindi%20Mukorossi%20Semen&qsr=herb_en_name&token=fb321389429665aba69eb20f7267aa39) | NO |  |  |  |  |
| Lignoceric acid | [Sapindi Mukorossi Semen](http://tcmspw.com/tcmspsearch.php?qr=Sapindi%20Mukorossi%20Semen&qsr=herb_en_name&token=fb321389429665aba69eb20f7267aa39) | NO |  |  |  |  |
| (2R)-2-[(3S,5R,10S,13R,14R,16R,17R)-3,16-dihydroxy-4,4,10,13,14-pentamethyl-2,3,5,6,12,15,16,17-octahydro-1H-cyclopenta[a]phenanthren-17-yl]-6-methylhept-5-enoic acid | [Poria Cocos (Schw.) Wolf.](http://tcmspw.com/tcmspsearch.php?qr=Poria%20Cocos(Schw.)%20Wolf.&qsr=herb_en_name&token=fb321389429665aba69eb20f7267aa39) | NO |  |  |  |  |
| Trametenolic acid | [Poria Cocos (Schw.) Wolf.](http://tcmspw.com/tcmspsearch.php?qr=Poria%20Cocos(Schw.)%20Wolf.&qsr=herb_en_name&token=fb321389429665aba69eb20f7267aa39) | YES |  |  | inhibitition | PMID:25197307 |
| Cerevisterol | [Poria Cocos (Schw.) Wolf.](http://tcmspw.com/tcmspsearch.php?qr=Poria%20Cocos(Schw.)%20Wolf.&qsr=herb_en_name&token=fb321389429665aba69eb20f7267aa39) | NO |  |  |  |  |
| Ergosta-7,22E-dien-3beta-ol | [Poria Cocos (Schw.) Wolf.](http://tcmspw.com/tcmspsearch.php?qr=Poria%20Cocos(Schw.)%20Wolf.&qsr=herb_en_name&token=fb321389429665aba69eb20f7267aa39) | NO |  |  |  |  |
| Ergosterol peroxide | [Poria Cocos (Schw.) Wolf.](http://tcmspw.com/tcmspsearch.php?qr=Poria%20Cocos(Schw.)%20Wolf.&qsr=herb_en_name&token=fb321389429665aba69eb20f7267aa39) | YES |  |  | inhibitition | PMID:20134236 |
| β-amyrin acetate | [Poria Cocos (Schw.) Wolf.](http://tcmspw.com/tcmspsearch.php?qr=Poria%20Cocos(Schw.)%20Wolf.&qsr=herb_en_name&token=fb321389429665aba69eb20f7267aa39) | YES |  |  | inhibitition | PMID:23080382 |
| Octacosane | [Paeoniae Radix Alba](http://tcmspw.com/tcmspsearch.php?qr=Paeoniae%20Radix%20Alba&qsr=herb_en_name&token=fb321389429665aba69eb20f7267aa39) | NO |  |  |  |  |
| Astragalin | [Paeoniae Radix Alba](http://tcmspw.com/tcmspsearch.php?qr=Paeoniae%20Radix%20Alba&qsr=herb_en_name&token=fb321389429665aba69eb20f7267aa39) | YES |  |  | inhibitition | PMID:25898764 |
| (3S,5R,8R,9R,10S,14S)-3,17-dihydroxy-4,4,8,10,14-pentamethyl-2,3,5,6,7,9-hexahydro-1H-cyclopenta[a]phenanthrene-15,16-dione | [Paeoniae Radix Alba](http://tcmspw.com/tcmspsearch.php?qr=Paeoniae%20Radix%20Alba&qsr=herb_en_name&token=fb321389429665aba69eb20f7267aa39) | NO |  |  |  |  |
| [(3S,3aR,6S,7aR)-6-hydroxy-6-methyl-2,5-dioxo-3a,4,7,7a-tetrahydro-3H-benzofuran-3-yl] methyl benzoate | [Paeoniae Radix Alba](http://tcmspw.com/tcmspsearch.php?qr=Paeoniae%20Radix%20Alba&qsr=herb_en_name&token=fb321389429665aba69eb20f7267aa39) | NO |  |  |  |  |
| Paeoniflorin | [Paeoniae Radix Alba](http://tcmspw.com/tcmspsearch.php?qr=Paeoniae%20Radix%20Alba&qsr=herb_en_name&token=fb321389429665aba69eb20f7267aa39) | YES |  |  | Promotion | PMID:31704261 |
| 14-acetyl-12-senecioyl-2E,8Z,10E-atractylentriol | [Atractylodes Macrocephala Koidz.](http://tcmspw.com/tcmspsearch.php?qr=Atractylodes%20Macrocephala%20Koidz.&qsr=herb_en_name&token=fb321389429665aba69eb20f7267aa39) | NO |  |  |  |  |
| Stigmast-22E-en-3beta-ol | [Atractylodes Macrocephala Koidz.](http://tcmspw.com/tcmspsearch.php?qr=Atractylodes%20Macrocephala%20Koidz.&qsr=herb_en_name&token=fb321389429665aba69eb20f7267aa39) | NO |  |  |  |  |
| Biatractylolide | [Atractylodes Macrocephala Koidz.](http://tcmspw.com/tcmspsearch.php?qr=Atractylodes%20Macrocephala%20Koidz.&qsr=herb_en_name&token=fb321389429665aba69eb20f7267aa39) | NO |  |  |  |  |
| ATRACTYLODES MACROCEPHALA | [Atractylodes Macrocephala Koidz.](http://tcmspw.com/tcmspsearch.php?qr=Atractylodes%20Macrocephala%20Koidz.&qsr=herb_en_name&token=fb321389429665aba69eb20f7267aa39) | NO |  |  |  |  |
| 8β-ethoxy atractylenolide Ⅲ | [Atractylodes Macrocephala Koidz.](http://tcmspw.com/tcmspsearch.php?qr=Atractylodes%20Macrocephala%20Koidz.&qsr=herb_en_name&token=fb321389429665aba69eb20f7267aa39) | NO |  |  |  |  |
| 1,6-bis(p-hydroxybenzyl)-4-methoxy-9,10-dihydrophenanthrene | [Bletilla Striata (Thunb.Ex A. Murray) Rchb.F.](http://tcmspw.com/tcmspsearch.php?qr=Bletilla%20Striata%20(Thunb.Ex%20A.Murray)Rchb.F.&qsr=herb_en_name&token=fb321389429665aba69eb20f7267aa39) | NO |  |  |  |  |
| 1,8-bis(hydroxybenzyl)-4-methoxyphenanthrene-2,7-diol | [Bletilla Striata (Thunb.Ex A. Murray) Rchb.F.](http://tcmspw.com/tcmspsearch.php?qr=Bletilla%20Striata%20(Thunb.Ex%20A.Murray)Rchb.F.&qsr=herb_en_name&token=fb321389429665aba69eb20f7267aa39) | NO |  |  |  |  |
| 1-(4-hydroxybenzyl)-4-methoxy-9,10-dihydrophenanthrene-2,7-diol | [Bletilla Striata (Thunb.Ex A. Murray) Rchb.F.](http://tcmspw.com/tcmspsearch.php?qr=Bletilla%20Striata%20(Thunb.Ex%20A.Murray)Rchb.F.&qsr=herb_en_name&token=fb321389429665aba69eb20f7267aa39) | NO |  |  |  |  |
| 2,3,4,7-tetramethoxyphenanthrene | [Bletilla Striata (Thunb.Ex A. Murray) Rchb.F.](http://tcmspw.com/tcmspsearch.php?qr=Bletilla%20Striata%20(Thunb.Ex%20A.Murray)Rchb.F.&qsr=herb_en_name&token=fb321389429665aba69eb20f7267aa39) | NO |  |  |  |  |
| 2,4,7-trimethoxy-9,10-dihydrophenanthrene | [Bletilla Striata (Thunb.Ex A. Murray) Rchb.F.](http://tcmspw.com/tcmspsearch.php?qr=Bletilla%20Striata%20(Thunb.Ex%20A.Murray)Rchb.F.&qsr=herb_en_name&token=fb321389429665aba69eb20f7267aa39) | NO |  |  |  |  |
| 2,7-dihydroxy-4-methoxy-3-(p-hydroxybenzyl)-4-methoxy-9,10-dihydrophenanthrene | [Bletilla Striata (Thunb.Ex A. Murray) Rchb.F.](http://tcmspw.com/tcmspsearch.php?qr=Bletilla%20Striata%20(Thunb.Ex%20A.Murray)Rchb.F.&qsr=herb_en_name&token=fb321389429665aba69eb20f7267aa39) | NO |  |  |  |  |
| 2,4-bis(4-hydroxybenzyl)-3-[2-(3-hydroxyphenyl) ethyl]-5-methoxy-phenol | [Bletilla Striata (Thunb.Ex A. Murray) Rchb.F.](http://tcmspw.com/tcmspsearch.php?qr=Bletilla%20Striata%20(Thunb.Ex%20A.Murray)Rchb.F.&qsr=herb_en_name&token=fb321389429665aba69eb20f7267aa39) | NO |  |  |  |  |
| 3,7-dihydroxy-2,4-dimethoxyphenanthrene-3-O-glucoside | [Bletilla Striata (Thunb.Ex A. Murray) Rchb.F.](http://tcmspw.com/tcmspsearch.php?qr=Bletilla%20Striata%20(Thunb.Ex%20A.Murray)Rchb.F.&qsr=herb_en_name&token=fb321389429665aba69eb20f7267aa39) | NO |  |  |  |  |
| 4,7-dihydroxy-1-p-hydroxybenzyl-2-methoxy-9,10-dihydrophenanthrene | [Bletilla Striata (Thunb.Ex A. Murray) Rchb.F.](http://tcmspw.com/tcmspsearch.php?qr=Bletilla%20Striata%20(Thunb.Ex%20A.Murray)Rchb.F.&qsr=herb_en_name&token=fb321389429665aba69eb20f7267aa39) | NO |  |  |  |  |
| 7-methoxy-9,10-dihydrophenanthrene-2,5-diol | [Bletilla Striata (Thunb.Ex A. Murray) Rchb.F.](http://tcmspw.com/tcmspsearch.php?qr=Bletilla%20Striata%20(Thunb.Ex%20A.Murray)Rchb.F.&qsr=herb_en_name&token=fb321389429665aba69eb20f7267aa39) | NO |  |  |  |  |
| Bletlol B | [Bletilla Striata (Thunb.Ex A. Murray) Rchb.F.](http://tcmspw.com/tcmspsearch.php?qr=Bletilla%20Striata%20(Thunb.Ex%20A.Murray)Rchb.F.&qsr=herb_en_name&token=fb321389429665aba69eb20f7267aa39) | NO |  |  |  |  |
| Bletlol C | [Bletilla Striata (Thunb.Ex A. Murray) Rchb.F.](http://tcmspw.com/tcmspsearch.php?qr=Bletilla%20Striata%20(Thunb.Ex%20A.Murray)Rchb.F.&qsr=herb_en_name&token=fb321389429665aba69eb20f7267aa39) | NO |  |  |  |  |
| Blespirol | [Bletilla Striata (Thunb.Ex A. Murray) Rchb.F.](http://tcmspw.com/tcmspsearch.php?qr=Bletilla%20Striata%20(Thunb.Ex%20A.Murray)Rchb.F.&qsr=herb_en_name&token=fb321389429665aba69eb20f7267aa39) | NO |  |  |  |  |
| 1-(2,7-dihydroxy-4-methoxy-9,10-dihydrophenanthren-1-yl)-4-methoxy-9,10-dihydrophenanthrene-2,7-diol | [Bletilla Striata (Thunb.Ex A. Murray) Rchb.F.](http://tcmspw.com/tcmspsearch.php?qr=Bletilla%20Striata%20(Thunb.Ex%20A.Murray)Rchb.F.&qsr=herb_en_name&token=fb321389429665aba69eb20f7267aa39) | NO |  |  |  |  |
| Blestrianol A | [Bletilla Striata (Thunb.Ex A. Murray) Rchb.F.](http://tcmspw.com/tcmspsearch.php?qr=Bletilla%20Striata%20(Thunb.Ex%20A.Murray)Rchb.F.&qsr=herb_en_name&token=fb321389429665aba69eb20f7267aa39) | NO |  |  |  |  |
| Blestrin A | [Bletilla Striata (Thunb.Ex A. Murray) Rchb.F.](http://tcmspw.com/tcmspsearch.php?qr=Bletilla%20Striata%20(Thunb.Ex%20A.Murray)Rchb.F.&qsr=herb_en_name&token=fb321389429665aba69eb20f7267aa39) | NO |  |  |  |  |
| Blestrin B | [Bletilla Striata (Thunb.Ex A. Murray) Rchb.F.](http://tcmspw.com/tcmspsearch.php?qr=Bletilla%20Striata%20(Thunb.Ex%20A.Murray)Rchb.F.&qsr=herb_en_name&token=fb321389429665aba69eb20f7267aa39) | NO |  |  |  |  |
| Blestrin C | [Bletilla Striata (Thunb.Ex A. Murray) Rchb.F.](http://tcmspw.com/tcmspsearch.php?qr=Bletilla%20Striata%20(Thunb.Ex%20A.Murray)Rchb.F.&qsr=herb_en_name&token=fb321389429665aba69eb20f7267aa39) | NO |  |  |  |  |
| Blestrin D | [Bletilla Striata (Thunb.Ex A. Murray) Rchb.F.](http://tcmspw.com/tcmspsearch.php?qr=Bletilla%20Striata%20(Thunb.Ex%20A.Murray)Rchb.F.&qsr=herb_en_name&token=fb321389429665aba69eb20f7267aa39) | NO |  |  |  |  |
| Crysophanol | [Ampelopsis Japonica](http://tcmspw.com/tcmspsearch.php?qr=Ampelopsis%20Japonica&qsr=herb_en_name&token=fb321389429665aba69eb20f7267aa39) | NO |  |  |  |  |
| (2R,3R,4S)-4-(4-hydroxy-3-methoxy-phenyl)-7-methoxy-2,3-dimethylol-tetralin-6-ol | [Ampelopsis Japonica](http://tcmspw.com/tcmspsearch.php?qr=Ampelopsis%20Japonica&qsr=herb_en_name&token=fb321389429665aba69eb20f7267aa39) | NO |  |  |  |  |
| Spinasterol | [Ampelopsis Japonica](http://tcmspw.com/tcmspsearch.php?qr=Ampelopsis%20Japonica&qsr=herb_en_name&token=fb321389429665aba69eb20f7267aa39) | YES |  |  | unclear | PMID:31370334 |
| Emodin | [Ampelopsis Japonica](http://tcmspw.com/tcmspsearch.php?qr=Ampelopsis%20Japonica&qsr=herb_en_name&token=fb321389429665aba69eb20f7267aa39) | YES |  |  | inhibitition | PMID:19585486 |
| Digallate | [Ampelopsis Japonica](http://tcmspw.com/tcmspsearch.php?qr=Ampelopsis%20Japonica&qsr=herb_en_name&token=fb321389429665aba69eb20f7267aa39) |  |  | 3'-digallate | inhibitition | PMID:19502752 |
| G6657_SIGMA | [Ampelopsis Japonica](http://tcmspw.com/tcmspsearch.php?qr=Ampelopsis%20Japonica&qsr=herb_en_name&token=fb321389429665aba69eb20f7267aa39) | NO |  |  |  |  |
| (-)-Catechin gallate | [Ampelopsis Japonica](http://tcmspw.com/tcmspsearch.php?qr=Ampelopsis%20Japonica&qsr=herb_en_name&token=fb321389429665aba69eb20f7267aa39) |  |  | epigallocatechin gallate | inhibitition | PMID:25643794 |
| Quercitrin | [Ampelopsis Japonica](http://tcmspw.com/tcmspsearch.php?qr=Ampelopsis%20Japonica&qsr=herb_en_name&token=fb321389429665aba69eb20f7267aa39) | YES |  |  | inhibitition | PMID:15388977 |
| Ent-Epicatechin | [Ampelopsis Japonica](http://tcmspw.com/tcmspsearch.php?qr=Ampelopsis%20Japonica&qsr=herb_en_name&token=fb321389429665aba69eb20f7267aa39) |  |  | epigallocatechin gallate | inhibitition | PMID:25643794 |
| HEXACOSANE | [A. Dahurica (Fisch.) Benth. Et Hook](http://tcmspw.com/tcmspsearch.php?qr=A.%20Dahurica%20(Fisch.)%20Benth.%20Et%20Hook&qsr=herb_en_name&token=fb321389429665aba69eb20f7267aa39) | NO |  |  |  |  |
| Alloisoimperatorin | [A. Dahurica (Fisch.) Benth. Et Hook](http://tcmspw.com/tcmspsearch.php?qr=A.%20Dahurica%20(Fisch.)%20Benth.%20Et%20Hook&qsr=herb_en_name&token=fb321389429665aba69eb20f7267aa39) | NO |  |  |  |  |
| Ammidin | [A. Dahurica (Fisch.) Benth. Et Hook](http://tcmspw.com/tcmspsearch.php?qr=A.%20Dahurica%20(Fisch.)%20Benth.%20Et%20Hook&qsr=herb_en_name&token=fb321389429665aba69eb20f7267aa39) | NO |  |  |  |  |
| Isoimperatorin | [A. Dahurica (Fisch.) Benth. Et Hook](http://tcmspw.com/tcmspsearch.php?qr=A.%20Dahurica%20(Fisch.)%20Benth.%20Et%20Hook&qsr=herb_en_name&token=fb321389429665aba69eb20f7267aa39) | YES |  |  | inhibitition | PMID:32299303 |
| Cnidilin | [A. Dahurica (Fisch.) Benth. Et Hook](http://tcmspw.com/tcmspsearch.php?qr=A.%20Dahurica%20(Fisch.)%20Benth.%20Et%20Hook&qsr=herb_en_name&token=fb321389429665aba69eb20f7267aa39) | NO |  |  |  |  |
| Byakangelicin | [A. Dahurica (Fisch.) Benth. Et Hook](http://tcmspw.com/tcmspsearch.php?qr=A.%20Dahurica%20(Fisch.)%20Benth.%20Et%20Hook&qsr=herb_en_name&token=fb321389429665aba69eb20f7267aa39) | YES |  |  | Promotion | PMID:28754032 |
| Neobyakangelico l | [A. Dahurica (Fisch.) Benth. Et Hook](http://tcmspw.com/tcmspsearch.php?qr=A.%20Dahurica%20(Fisch.)%20Benth.%20Et%20Hook&qsr=herb_en_name&token=fb321389429665aba69eb20f7267aa39) | NO |  |  |  |  |
| 5-[[(2S)-3,3-dimethyloxiran-2-yl] methoxy]-3,7-dihydropyrano[3,2-f] benzofuran-2-one | [A. Dahurica (Fisch.) Benth. Et Hook](http://tcmspw.com/tcmspsearch.php?qr=A.%20Dahurica%20(Fisch.)%20Benth.%20Et%20Hook&qsr=herb_en_name&token=fb321389429665aba69eb20f7267aa39) | NO |  |  |  |  |
| {5-[2'(R)-Hydroxy-3'-methyl-3'-butenyl-oxy] furocoumarin} | [A. Dahurica (Fisch.) Benth. Et Hook](http://tcmspw.com/tcmspsearch.php?qr=A.%20Dahurica%20(Fisch.)%20Benth.%20Et%20Hook&qsr=herb_en_name&token=fb321389429665aba69eb20f7267aa39) | NO |  |  |  |  |
| 9-[[(2R)-3,3-dimethyloxiran-2-yl] methoxy] furo[3,2-g]chromen-7-one | [A. Dahurica (Fisch.) Benth. Et Hook](http://tcmspw.com/tcmspsearch.php?qr=A.%20Dahurica%20(Fisch.)%20Benth.%20Et%20Hook&qsr=herb_en_name&token=fb321389429665aba69eb20f7267aa39) | NO |  |  |  |  |
| Byakangelicol | [A. Dahurica (Fisch.) Benth. Et Hook](http://tcmspw.com/tcmspsearch.php?qr=A.%20Dahurica%20(Fisch.)%20Benth.%20Et%20Hook&qsr=herb_en_name&token=fb321389429665aba69eb20f7267aa39) | NO |  |  |  |  |
| Pyrene | [A. Dahurica (Fisch.) Benth. Et Hook](http://tcmspw.com/tcmspsearch.php?qr=A.%20Dahurica%20(Fisch.)%20Benth.%20Et%20Hook&qsr=herb_en_name&token=fb321389429665aba69eb20f7267aa39) |  |  | Benzo[a]pyrene | inhibitition | PMID:29477119 |
| 4-[(2S)-2,3-dihydroxy-3-methylbutoxy] furo[3,2-g] chromen-7-one | [A. Dahurica (Fisch.) Benth. Et Hook](http://tcmspw.com/tcmspsearch.php?qr=A.%20Dahurica%20(Fisch.)%20Benth.%20Et%20Hook&qsr=herb_en_name&token=fb321389429665aba69eb20f7267aa39) | NO |  |  |  |  |
| Sen-byakangelicol | [A. Dahurica (Fisch.) Benth. Et Hook](http://tcmspw.com/tcmspsearch.php?qr=A.%20Dahurica%20(Fisch.)%20Benth.%20Et%20Hook&qsr=herb_en_name&token=fb321389429665aba69eb20f7267aa39) | NO |  |  |  |  |
| Tetracosane | [A. Dahurica (Fisch.) Benth. Et Hook](http://tcmspw.com/tcmspsearch.php?qr=A.%20Dahurica%20(Fisch.)%20Benth.%20Et%20Hook&qsr=herb_en_name&token=fb321389429665aba69eb20f7267aa39) | NO |  |  |  |  |
| ZINC03860434 | [A. Dahurica (Fisch.) Benth. Et Hook](http://tcmspw.com/tcmspsearch.php?qr=A.%20Dahurica%20(Fisch.)%20Benth.%20Et%20Hook&qsr=herb_en_name&token=fb321389429665aba69eb20f7267aa39) | NO |  |  |  |  |
| Phellopterin | [A. Dahurica (Fisch.) Benth. Et Hook](http://tcmspw.com/tcmspsearch.php?qr=A.%20Dahurica%20(Fisch.)%20Benth.%20Et%20Hook&qsr=herb_en_name&token=fb321389429665aba69eb20f7267aa39) | YES |  |  | unclear | PMID:31510069 |
| Prangenidin | [A. Dahurica (Fisch.) Benth. Et Hook](http://tcmspw.com/tcmspsearch.php?qr=A.%20Dahurica%20(Fisch.)%20Benth.%20Et%20Hook&qsr=herb_en_name&token=fb321389429665aba69eb20f7267aa39) | NO |  |  |  |  |
| Linolein, 2-mono- | [A. Dahurica (Fisch.) Benth. Et Hook](http://tcmspw.com/tcmspsearch.php?qr=A.%20Dahurica%20(Fisch.)%20Benth.%20Et%20Hook&qsr=herb_en_name&token=fb321389429665aba69eb20f7267aa39) | NO |  |  |  |  |
| Osmanthuside H | [A. Dahurica (Fisch.) Benth. Et Hook](http://tcmspw.com/tcmspsearch.php?qr=A.%20Dahurica%20(Fisch.)%20Benth.%20Et%20Hook&qsr=herb_en_name&token=fb321389429665aba69eb20f7267aa39) | NO |  |  |  |  |
| Prangenin | [A. Dahurica (Fisch.) Benth. Et Hook](http://tcmspw.com/tcmspsearch.php?qr=A.%20Dahurica%20(Fisch.)%20Benth.%20Et%20Hook&qsr=herb_en_name&token=fb321389429665aba69eb20f7267aa39) | NO |  |  |  |  |
| Wogonin | [Atractylodes Lancea (Thunb.) Dc.](http://tcmspw.com/tcmspsearch.php?qr=Atractylodes%20Lancea%20(Thunb.)Dc.&qsr=herb_en_name&token=fb321389429665aba69eb20f7267aa39) | YES |  |  | inhibitition | PMID:29104663 |
| NSC63551 | [Atractylodes Lancea (Thunb.) Dc.](http://tcmspw.com/tcmspsearch.php?qr=Atractylodes%20Lancea%20(Thunb.)Dc.&qsr=herb_en_name&token=fb321389429665aba69eb20f7267aa39) | NO |  |  |  |  |
| Stigmasterol 3-O-beta-D-glucopyranoside | [Atractylodes Lancea (Thunb.) Dc.](http://tcmspw.com/tcmspsearch.php?qr=Atractylodes%20Lancea%20(Thunb.)Dc.&qsr=herb_en_name&token=fb321389429665aba69eb20f7267aa39) |  |  | Stigmasterol | unclear | PMID: 23891889 |
| 3β-acetoxyatractylone | [Atractylodes Lancea (Thunb.) Dc.](http://tcmspw.com/tcmspsearch.php?qr=Atractylodes%20Lancea%20(Thunb.)Dc.&qsr=herb_en_name&token=fb321389429665aba69eb20f7267aa39) | NO |  |  |  |  |
| 3,5-dimethoxy-4-glucosyloxyphenylallylalcohol | [Atractylodes Lancea (Thunb.) Dc.](http://tcmspw.com/tcmspsearch.php?qr=Atractylodes%20Lancea%20(Thunb.)Dc.&qsr=herb_en_name&token=fb321389429665aba69eb20f7267aa39) | NO |  |  |  |  |
| Beta-daucosterol | [Atractylodes Lancea (Thunb.) Dc.](http://tcmspw.com/tcmspsearch.php?qr=Atractylodes%20Lancea%20(Thunb.)Dc.&qsr=herb_en_name&token=fb321389429665aba69eb20f7267aa39) |  |  | daucosterol | unclear | PMID:22468750 |
| Beta-daucosterol_qt | [Atractylodes Lancea (Thunb.) Dc.](http://tcmspw.com/tcmspsearch.php?qr=Atractylodes%20Lancea%20(Thunb.)Dc.&qsr=herb_en_name&token=fb321389429665aba69eb20f7267aa39) |  |  | daucosterol | unclear | PMID:22468750 |
| (24S)-5beta-Stigmastan-3beta-ol | [Atractylodes Lancea (Thunb.) Dc.](http://tcmspw.com/tcmspsearch.php?qr=Atractylodes%20Lancea%20(Thunb.)Dc.&qsr=herb_en_name&token=fb321389429665aba69eb20f7267aa39) | NO |  |  |  |  |
| beta-sitosterol 3-O-glucoside | [Atractylodes Lancea (Thunb.) Dc.](http://tcmspw.com/tcmspsearch.php?qr=Atractylodes%20Lancea%20(Thunb.)Dc.&qsr=herb_en_name&token=fb321389429665aba69eb20f7267aa39) |  |  | beta-sitosterol | inhibitition | PMID:31763737 |
| Daucosterin | [Atractylodes Lancea (Thunb.) Dc.](http://tcmspw.com/tcmspsearch.php?qr=Atractylodes%20Lancea%20(Thunb.)Dc.&qsr=herb_en_name&token=fb321389429665aba69eb20f7267aa39) | YES |  |  | unclear | PMID:16595931 |
| Delta 7-stigmastenol | [Atractylodes Lancea (Thunb.) Dc.](http://tcmspw.com/tcmspsearch.php?qr=Atractylodes%20Lancea%20(Thunb.)Dc.&qsr=herb_en_name&token=fb321389429665aba69eb20f7267aa39) |  |  | stigmastenol | unclear | PMID: 23891889 |
| Oleanolic acid-28-O-beta-D-glucopyranoside | [Fructus Ligustri Lucidi](http://tcmspw.com/tcmspsearch.php?qr=Fructus%20Ligustri%20Lucidi&qsr=herb_en_name&token=fb321389429665aba69eb20f7267aa39) |  |  | oleanolic acid 3-O-β-D-glucopyranosyl (1→3)-β-D-glucopyranosiduronic acid | Promotion | PMID;26887328 |
| (-)-Olivir | [Fructus Ligustri Lucidi](http://tcmspw.com/tcmspsearch.php?qr=Fructus%20Ligustri%20Lucidi&qsr=herb_en_name&token=fb321389429665aba69eb20f7267aa39) | NO |  |  |  |  |
| Acteoside | [Fructus Ligustri Lucidi](http://tcmspw.com/tcmspsearch.php?qr=Fructus%20Ligustri%20Lucidi&qsr=herb_en_name&token=fb321389429665aba69eb20f7267aa39) | YES |  |  | inhibitition | PMID:21899547 |
| Salidroside | [Fructus Ligustri Lucidi](http://tcmspw.com/tcmspsearch.php?qr=Fructus%20Ligustri%20Lucidi&qsr=herb_en_name&token=fb321389429665aba69eb20f7267aa39) | YES |  |  | inhibitition | PMID:23746955 |
| Taxifolin | [Fructus Ligustri Lucidi](http://tcmspw.com/tcmspsearch.php?qr=Fructus%20Ligustri%20Lucidi&qsr=herb_en_name&token=fb321389429665aba69eb20f7267aa39) | YES |  |  | inhibitition | PMID:18729255 |
| Ursolic acid | [Fructus Ligustri Lucidi](http://tcmspw.com/tcmspsearch.php?qr=Fructus%20Ligustri%20Lucidi&qsr=herb_en_name&token=fb321389429665aba69eb20f7267aa39) | YES |  |  | inhibitition | PMID:28492484 |
| Lucidumoside D_qt | [Fructus Ligustri Lucidi](http://tcmspw.com/tcmspsearch.php?qr=Fructus%20Ligustri%20Lucidi&qsr=herb_en_name&token=fb321389429665aba69eb20f7267aa39) | NO |  |  |  |  |
| Tormentic acid | [Fructus Ligustri Lucidi](http://tcmspw.com/tcmspsearch.php?qr=Fructus%20Ligustri%20Lucidi&qsr=herb_en_name&token=fb321389429665aba69eb20f7267aa39) | NO |  |  |  |  |
| Eriodictyol | [Fructus Ligustri Lucidi](http://tcmspw.com/tcmspsearch.php?qr=Fructus%20Ligustri%20Lucidi&qsr=herb_en_name&token=fb321389429665aba69eb20f7267aa39) | YES |  |  | inhibitition | PMID:26141996 |
| Olitoriside_qt | [Fructus Ligustri Lucidi](http://tcmspw.com/tcmspsearch.php?qr=Fructus%20Ligustri%20Lucidi&qsr=herb_en_name&token=fb321389429665aba69eb20f7267aa39) | NO |  |  |  |  |
| Luteolin | [Fructus Ligustri Lucidi](http://tcmspw.com/tcmspsearch.php?qr=Fructus%20Ligustri%20Lucidi&qsr=herb_en_name&token=fb321389429665aba69eb20f7267aa39) | YES |  |  | inhibitition | PMID:28753056 |
| Cosmetin | [Fructus Ligustri Lucidi](http://tcmspw.com/tcmspsearch.php?qr=Fructus%20Ligustri%20Lucidi&qsr=herb_en_name&token=fb321389429665aba69eb20f7267aa39) | NO |  |  |  |  |
| Apigenin | [Fructus Ligustri Lucidi](http://tcmspw.com/tcmspsearch.php?qr=Fructus%20Ligustri%20Lucidi&qsr=herb_en_name&token=fb321389429665aba69eb20f7267aa39) | YES |  |  | Promotion | PMID:20638260 |
| Luteolin-7-o-glucoside | [Fructus Ligustri Lucidi](http://tcmspw.com/tcmspsearch.php?qr=Fructus%20Ligustri%20Lucidi&qsr=herb_en_name&token=fb321389429665aba69eb20f7267aa39) |  | Malcolmia littorea (L.) R.Br. Extracts (salicylic acid and luteolin-7-O-glucoside) |  | inhibitition | PMID:32013559 |
| Sitosterol alpha1 | [Coicis Semen](http://tcmspw.com/tcmspsearch.php?qr=Coicis%20Semen&qsr=herb_en_name&token=fb321389429665aba69eb20f7267aa39) |  |  | beta-sitosterol | inhibitition | PMID:31763737 |
| Isoarborinol | [Coicis Semen](http://tcmspw.com/tcmspsearch.php?qr=Coicis%20Semen&qsr=herb_en_name&token=fb321389429665aba69eb20f7267aa39) | NO |  |  |  |  |
| Omaine | [Coicis Semen](http://tcmspw.com/tcmspsearch.php?qr=Coicis%20Semen&qsr=herb_en_name&token=fb321389429665aba69eb20f7267aa39) | NO |  |  |  |  |
| Campesterol | [Coicis Semen](http://tcmspw.com/tcmspsearch.php?qr=Coicis%20Semen&qsr=herb_en_name&token=fb321389429665aba69eb20f7267aa39) |  | aqueous decoction (AD) and methanolic extract (ME) of roots of C. opaca |  | inhibitition | PMID:29142412 |
