# Supplementary material for: Identification of Sitogluside as a Potential Skin-Pigmentation-Reducing Agent through Network Pharmacology
Source: Oxid Med Cell Longev. 2021 Sep 23;2021:4883398. doi: 10.1155/2021/4883398 (PMC8483913; doi:10.1155/2021/4883398)
Supplement: Supplementary Materials — Figure S1: (A) expression of p-CREB, CREB, p-JUK, and JUK in MAPK and PKA pathways was determined by western blotting. (B) CCK8 assay detects the cell viability of MNT1 and B16F0 cells treated with different concentrations of PMA and anisomycin, respectively. (C) PMA can significantly upregulate the phosphorylation level of ERK in MNT1 and B16F0 cells. (D) Within eight hours, PMA significantly increased the phosphorylation level of ERK. (E) Anisomycin can significantly upregulate the phosphorylation level of p38 in MNT1 and B16F0 cells. (F) Within eight hours, anisomycin significantly increased the phosphorylation level of p38. Figure S2: (A, B) The statistical results of western blotting of function recovery experiment in MNT1 and B16F0. (C) A near-infrared fluorescence probe was used to detect the TYR activity in B16F0. Table S1: basic information of traditional Chinese medicine in Ben-Cao-Gang-Mu and folk prescriptions. Table S2: the Venn analysis of potential effective components of 5 traditional Chinese medicines. Table S3: details of the potential active ingredients of 14 traditional Chinese medicines. [file 4883398.f1.zip › supplementary Figure.doc]

**Fig. S1.**

**(A)** Expression of p-CREB, CREB, p-JUK and JUK in MAPK and PKA pathways was determined by western blotting. **(B)** CCK8 assay detects the cell viability of MNT1 and B16F0 cells treated with different concentrations of PMA and anisomycin, respectively. **(C)** PMA can significantly up-regulate the phosphorylation level of ERK in MNT1 and B16F0 cells. **(D)** Within eight hours, PMA significantly increased the phosphorylation level of ERK. **(E)** Anisomycin can significantly up-regulate the phosphorylation level of P38 in MNT1 and B16F0 cells. **(F)** Within eight hours, anisomycin significantly increased the phosphorylation level of P38.

**Fig. S2.**

(A-B) The statistical results of western blotting of function recovery experiment in MNT1 and B16F0. (C) A near-infrared fluorescence probe was used to detect the TYR activity in B16F0.
